# Does a Machine-Learned Potential Perform Better Than an Optimally Tuned Traditional Force Field? A Case Study on Fluorohydrins

**DOI:** 10.1021/acs.jcim.2c01510

**Published:** 2023-04-18

**Authors:** João Morado, Paul N. Mortenson, J. Willem M. Nissink, Jonathan W. Essex, Chris-Kriton Skylaris

**Affiliations:** † School of Chemistry, University of Southampton, Highfield, Southampton SO17 1BJ, United Kingdom; ‡ Astex Pharmaceuticals, 436 Cambridge Science Park, Milton Road, Cambridge CB4 0QA, United Kingdom; § Computational Chemistry, Oncology R&D, AstraZeneca, Cambridge CB4 0WG, United Kingdom

## Abstract

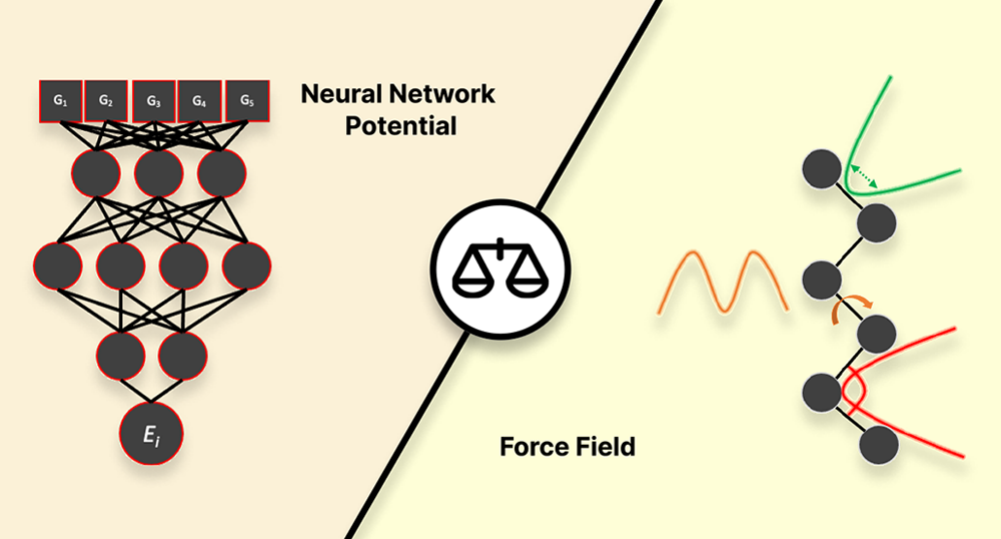

We present a comparative study that evaluates the performance of a machine learning potential (ANI-2x), a conventional force field (GAFF), and an optimally tuned GAFF-like force field in the modeling of a set of 10 γ-fluorohydrins that exhibit a complex interplay between intra- and intermolecular interactions in determining conformer stability. To benchmark the performance of each molecular model, we evaluated their energetic, geometric, and sampling accuracies relative to quantum-mechanical data. This benchmark involved conformational analysis both in the gas phase and chloroform solution. We also assessed the performance of the aforementioned molecular models in estimating nuclear spin–spin coupling constants by comparing their predictions to experimental data available in chloroform. The results and discussion presented in this study demonstrate that ANI-2x tends to predict stronger-than-expected hydrogen bonding and overstabilize global minima and shows problems related to inadequate description of dispersion interactions. Furthermore, while ANI-2x is a viable model for modeling in the gas phase, conventional force fields still play an important role, especially for condensed-phase simulations. Overall, this study highlights the strengths and weaknesses of each model, providing guidelines for the use and future development of force fields and machine learning potentials.

## 1 Introduction

Modeling of small organic molecules (hereafter termed ligands) is a key aspect of many disciplines in chemical sciences.^1^ Various areas of intensive research in the field, such as free energy calculations,^2−5^ molecular docking,^6,7^ and conformational analysis,^8,9^ require frequent modeling of different types of ligands. These applications are of paramount importance to the pharmaceutical industry because, when successfully applied, computational approaches can dramatically accelerate drug discovery. Lead optimization, in particular, can greatly benefit from rational analysis of the structural and energetics information that can be extracted from *in silico* experiments, which can produce either novel predictions or corroborate data obtained from other sources.^10−12^ These computational techniques have the advantage of being faster to perform than *in vitro* and *in vivo* experiments.^13^ They also have a substantially lower cost, making their optimization the way forward toward cheaper and more efficient drug discovery.^14^ It is therefore of the foremost importance to continue improving and benchmarking the current plethora of theoretical molecular models available to simulate ligands, since the accuracy of theoretical results ultimately relies on their performance.

Over the years, many methods have been developed to describe the potential energy of small organic molecules. Researchers have been attempting to find a compromise between accuracy and computational cost and a balance between the time scale that simulations can achieve and the size of the systems being simulated. Despite many efforts and advances toward this equilibrium, in general there is still a positive correlation between computational cost and accuracy, and these two properties are often negatively correlated with system size and simulation time scale. The gold-standard method in computational modeling remains to be quantum mechanics (QM). QM methods approximate the Schrödinger equation using wavefunction-based methods or density functional theory (DFT). From the wavefunction-based methods, the coupled-cluster methods^15,16^ are thus far the most accurate for quantum chemistry applications. Unfortunately, because of the high computational cost of QM methods, their use in *ab initio* simulations is limited to all but the simplest systems, despite recent advances that attempt to combine the sampling efficiency of cheap, approximate potentials with the accuracy of the quantum level.^17−21^ A less computationally demanding and widely employed alternative is the use of molecular mechanics (MM) force fields (FFs). FFs resort to classical empirical functions to describe the potential energy of systems. The popularity of FFs resides in their ability to simulate systems containing thousands of atoms on simulation time scales that can reach milliseconds.^22,23^ The main drawback of FFs comes from the same feature that confers them their strength: the simplicity of their functional form, while computationally attractive, is often unsuited to model complex chemistry and challenging chemical interactions, a constraint that is further aggravated by the requirement of a sometimes unknown set of FF parameters. Although FF parameters cover a wide range of classes of molecules at a satisfactory degree of accuracy, novel chemical entities, of which ligands are a striking example, frequently demand derivation of new FF parameters.^24−27^ The task of deriving FF parameters is known as FF parametrization, and for many applications it is neither trivial nor straightforward. Recently, kernel methods and neural network potentials (NNPs) have emerged as promising machine learning (ML) alternatives to FFs.^28−37^ NNPs learn the QM energy of an atom in its surrounding chemical environment, requiring neither an FF functional form nor FF parameters to work. For these reasons, NNPs are generally readily transferable to classes of compounds similar to those included in the training data set. The accuracy of NNPs when applied to chemical environments outside the training data set, however, is unpredictable and should be evaluated beforehand. Furthermore, although the computational cost of NNPs is much smaller than that of the QM methods (ca. 10^6^ times), even with GPU-accelerated computing they are still considerably more (up to 100 times) computationally expensive than conventional FFs, limiting the size of the systems that can be simulated and the simulation time scales that can be achieved.

**Figure 1:**
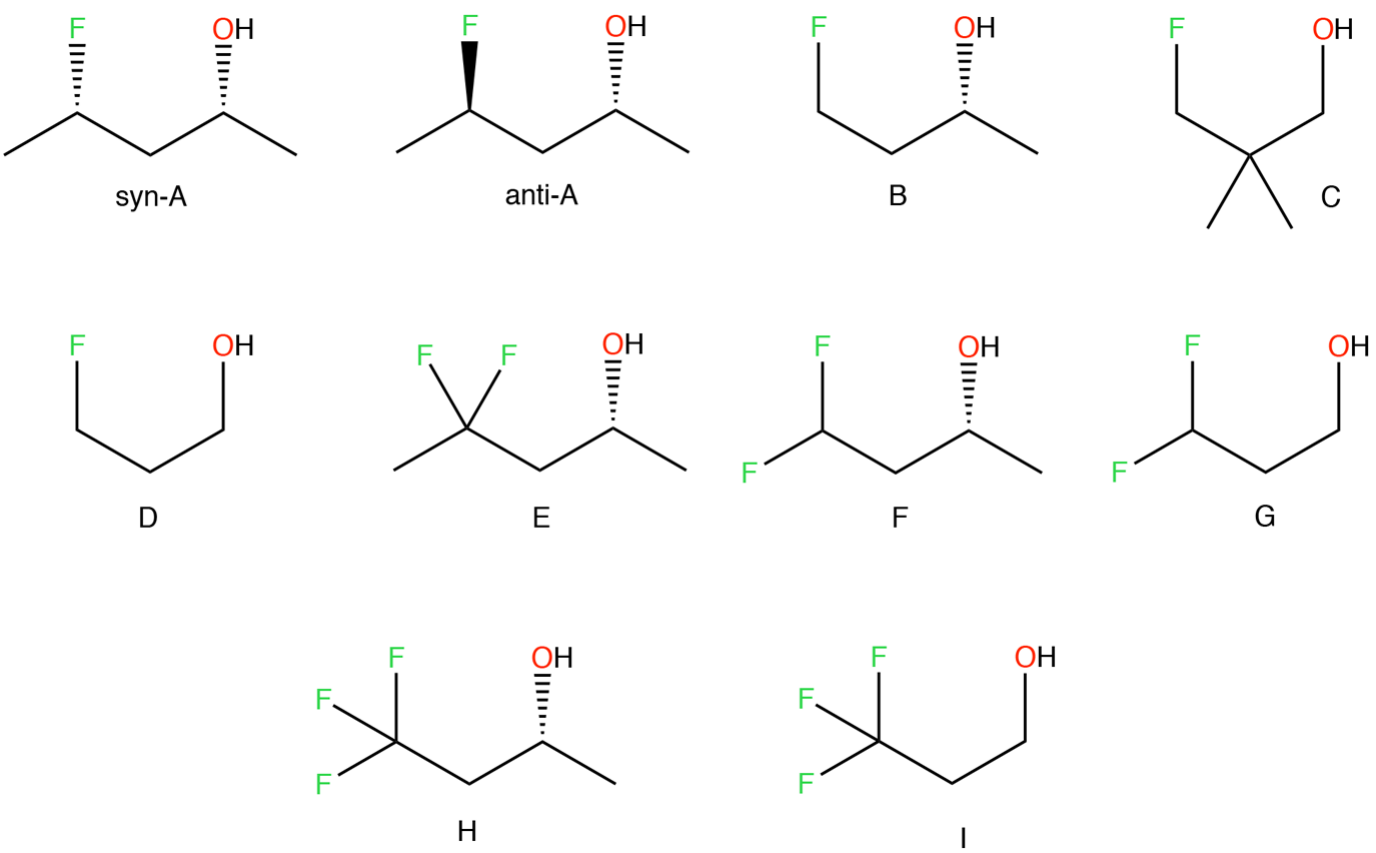
List of γ-fluorohydrins studied in this work.

In this article, we present results assessing the accuracy of an NNP, a conventional class I FF, and an optimally tuned FF in the modeling of a set of 10 γ-fluorohydrins (Figure 1). In 2015, Linclau et al. used these molecules to demonstrate for the first time the occurrence of OH–F intramolecular hydrogen bonds (IMHBs) in acyclic saturated γ-fluorohydrins.^38^ This set of molecules exhibits a complex interplay between intra- and intermolecular interactions in determining conformer stability, making it an interesting test case to study. We also benchmark the performance of the aforementioned molecular models both in the gas phase and chloroform solution by comparing their predictions to both experimental (NMR *J*-coupling constants) and theoretical data (energies and geometries).

The NNP we tested was ANI-2x,^39^ a model from the ANI family^35,40−43^ that has been trained to reproduce the ωB97X^44^/6-31G* level of theory. NNPs from the ANI family were designed to model neutral molecules in the singlet spin state, a limitation that has been recently circumvented by employing ML-based corrections to quantum methods.^45−48^ ANI-2x in specific was trained using a data set comprising 8.9 million molecular equilibrium and nonequilibrium conformations of fragments, and it includes active learning refinements to torsional profiles, nonbonded interactions, and bulk water behavior. This NNP has been the focus of various benchmarks and applications in recent years.^49−54^ Furthermore, as our traditional FF we used the General Amber FF (GAFF),^55^ which is commonly employed in the modeling of druglike molecules.^56^ Finally, our optimally tuned FF was an optimized version of the GAFF in which the bonded parameters were optimized to the same level of theory that ANI-2x was trained to reproduce (ωB97X/6-31G*).

Molecular dynamics (MD) was used to sample the molecular conformations. Since the NNP was used only to model the intramolecular interactions of the ligand, the simulations in chloroform employed a model that embeds the NNP inside a conventional MM FF. In this approach, the ligand intramolecular interactions are treated at the ML level, whereas the ligand–solvent intermolecular interactions and solvent–solvent interactions are treated using MM. This hybrid NNP/MM strategy excludes any polarization of the solute by the solvent and thus corresponds to a mechanical embedding model^57^ with fixed-point charges in the ML region. It has already been applied in past studies,^49,58−60^ being close in philosophy to quantum mechanics/molecular mechanics (QM/MM) models but with the NNP in place of the QM method.

This paper is structured as follows: we first describe the basic theory and methods underlying the present study, viz., the ANI-2x NNP, the FF reparametrization protocol, the hybrid NNP/MM model, the details regarding the MD simulations, and the procedure used to calculate the populations of conformers and the NMR *J*-coupling constants. We then present a thorough analysis of the performance of the tested molecular models both in the gas phase and in chloroform solution. Finally, we conclude with some final remarks and suggestions for future work.

## 2 Theory and Methods

### 2.1 The ANI-2x Neural Network Potential

NNPs are currently among the most promising potentials to be used in place of MM FFs to model the intramolecular interactions of ligands. There are currently two models of the ANI family that may be applied to a broad spectrum of problems in chemical sciences. ANI-1ccx, which is trained to reproduce CCSD(T*)/CBS, is the ANI NNP with the highest level of accuracy,^49,58,59,61^ although it can only simulate organic molecules containing elements H, C, N, and O. ANI-2x, on the other hand, is trained to reproduce ωB97X/6-31G* and has also shown promising results in some applications.^39,49,52^ ANI-2x has the advantage of extending the chemical space covered by ANI-1ccx to organic molecules also containing elements F, S, and Cl, the addition of which is essential for day-to-day use in common applications. Because of this, ANI-2x covers a chemical space that encompasses 90% of druglike molecules^39^ and is the only ANI model that can be employed to simulate the γ-fluorohydrins considered in this study due to the presence of the fluorine atoms.

The ANI-2x training data set was composed of molecules obtained from different sources, viz., the GDB-11^62,63^ and ChEMBL^64^ databases, the S66x8 benchmark,^65^ sulfur-containing amino acids and dipeptides randomly generated using RDKit.^66^ In total, 8.9 million molecular conformations were used. Fluorohydrins identical to or similar to those used in this study were part of the training data set, meaning that the ANI-2x model should be familiar with these types of molecular structures.

ANI NNPs overcome the requirement of an analytical FF functional form and a set of FF parameters by learning the QM energy of an atom *i*, *U*_*i*_, in its surrounding chemical environment. The sum of the individual atomic energies yields the total potential energy, *U*, of a given molecular species, i.e.,
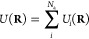
1in which *N*_a_ is the number of atoms of the system and **R** is a vector that maps a molecule into a certain mathematical representation, ideally invariant to translation and rotation. In terms of performance, the most encouraging feature of many ML models is that, once they have been trained, they can be applied to a myriad of systems without demanding the calculation of additional QM data. This yields the (nearly) linear scaling attributed to the ANI methods, which is bounded by the molecular featurization method employed to generate the descriptors that capture the atomic-local environment.^67^ As can be noted from eq 1, a molecular descriptor is the only input required by the NNPs to output the atomic energy. From the various flavors of available descriptors,^67−76^ ANI-2x uses the same atom-centered symmetry function as previous ANI models,^35^ viz., a form of Behler–Parrinello-type descriptors^77^ with a modified symmetry function for the angular part. The local environment approximation is introduced in these symmetry functions through cutoffs, which make ANI models unable to explicitly capture long-range effects. In this regard, a recent study showed that the poor long-range electrostatic description of ANI-2x has a deleterious effect on the prediction of water bulk properties (e.g., the internal pressure), even though this situation can be artificially compensated through the use of high external pressure values.^51^ Finally, regarding fitting to the ωB97X/6-31G* functional, the ANI-2x NNP was trained by minimizing the sum of the mean squared errors of the potential energies and forces.

### 2.2 Force Field Reparametrization

The conventional MM FF we used in this study was the GAFF,^55^ for which the functional form reads
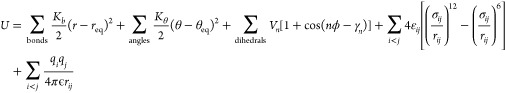
2in which *r*_eq_ and θ_eq_ are equilibrium structural parameters; *K*_*b*_, *K*_θ_, and *V*_*n*_ are the bond, angle, and dihedral force constants, respectively; *n* is the dihedral multiplicity and γ_*n*_ is the dihedral phase; ε_*ij*_ is the well depth of the Lennard-Jones (LJ) interaction between atoms *i* and *j* and σ_*ij*_ is the distance at which the said interaction vanishes; *q* is the atomic partial charge; and ϵ is the permittivity of free space. The partial charges were derived using the multiconformational restrained electrostatic potential (RESP) method.^78−80^ The gas-phase QM electrostatic potentials (ESPs) entering the RESP-fitting procedure were calculated at HF/6-31G*^80^ from gas-phase geometries optimized at ωB97X/6-31G*. The conformations used in these calculations were the major conformations found at the MP2/6-311++G(2d,p) level for each γ-fluorohydrin, as reported in ref (38). Two stages were performed in this charge-derivation process:^80−82^ first, the charges of all atoms were allowed to vary while hyperbolic regularization was applied with a scaling factor of 0.01; second, only the charges of the symmetry-equivalent H and F atoms were allowed to vary, and these charges were constrained to have the same value within a given symmetry group (in this stage, the scaling factor used for the hyperbolic regularization was 0.001).

The GAFF-like optimally tuned FFs, herein called GAFF.MOD, used the set of RESP charges previously derived and the same LJ parameters as in GAFF, but their bonded parameters (bonds, angles, and dihedral parameters) were further optimized to reproduce a training data set at ωB97X/6-31G*, the same level of theory that ANI-2x aims to reproduce. This training data set was composed of structures sampled from the DFT ensemble using the nested Markov chain Monte Carlo (nMC-MC) algorithm^83,84^ implemented in ParaMol,^26^ interfaced with Psi4^85^ for the QM calculations. nMC-MC combines sampling at an approximate potential with periodic switching attempts to the QM level, enabling recovery of the exact quantum DFT ensemble.^21^ Because of this feature, this method was used to generate high-quality structures representative of our reference level of theory. For each γ-fluorohydrin, a variable number of nMC-MC samplers were spawned, each starting from the major conformers reported in ref (38) (the same conformers used to calculate the ESPs for the RESP procedure). In total, each nMC-MC sampler performed 2.5 × 10^4^ sweeps, with hybrid Monte Carlo (hMC)^86^ runs of 100 steps carried out using a 1 fs time step. To accelerate sampling in the low-level chain (ANI-2x), a temperature of 350 K was used for its kinetic and potential energy terms, while the temperature of the target ωB97X/6-31G* *NVT* ensemble was 300 K. The collected nMC-MC data for each molecule were then merged, and from them training (1 × 10^4^ structures) and testing (3 × 10^4^ structures) data sets were generated by randomly collecting structures from the final DFT ensembles. The reparametrization of GAFF was finally conducted for each individual molecule by concomitantly optimizing all bond, angle, and dihedral parameters (except the dihedral phases) in eq 2. Only for molecule B, the dihedral phases (γ_*n*_ parameters in eq 2) also entered the optimization, as molecules containing chiral centers sometimes require adjustment of the dihedral phases to obtain a closer fit to the QM PES.^87,88^ Although some of the molecules represented in Figure 1 contain chiral centers, optimization of their dihedral phases only significantly improved the GAFF.MOD performance for molecule B. This was verified by running gas-phase simulations with both sets of parameters (with and without optimization of the dihedral phases) for each molecule, which showed that for all fluorohydrins except B, optimization of the dihedral phases led to similar or lower sampling accuracy or to breaking of important molecular symmetries (dihedral phases must be either 0° or 180° in order for individual conformational isomers to have the same dihedral energies). The parameter optimization was attained by minimizing the following objective function:
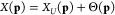
3in which **p** is the vector of FF parameters of a given molecule entering the optimization. Furthermore, *X*_*U*_ corresponds to the term of the objective function responsible for the fitting of the MM energies to QM data, given by
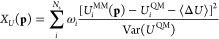
4in which *N*_*s*_ is the number of structures of a given molecule used in the parameter optimization, ω_*i*_ is the weight of the *i*th conformation (determined using the non-Boltzmann weighting method^26,89^), *U*_*i*_^QM^ and *U*_*i*_^MM^ are the QM and MM potential energies, Var(*U*^QM^) is the variance of the QM energies (here used as the normalization factor), and  is a term that brings the two distributions together by subtracting the average difference between the QM and MM potential energies from the energy residuals. Finally, Θ(**p**) is a regularization term included to prevent overfitting. In this work, we used a harmonic penalty function that assumes that the *prior* distribution of the *m*th parameter, *p*_*m*_, is a Gaussian centered at the initial guess *p*_*m*_^0^ with width γ_*m*_. The expression for this harmonic regularization term reads as
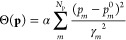
5in which α is an adjustable scaling factor that controls the strength of the regularization, here set to , in which *N*_p_ is the number of FF parameters entering the optimization. The values used for the *prior* widths can be found in Table S1. Both the RESP-fitting and reparametrization procedures were performed in ParaMol. ParaMol is freely available^26^ and greatly facilitates the process of parametrization of MM FFs.

### 2.3 The Hybrid Neural Network Potential/Molecular Mechanics Model

To determine the conformational dynamics of the set of γ-fluorohydrins considered in this study, we performed MD simulations both in the gas phase and in chloroform solution. For the simulations in chloroform that described the γ-fluorohydrins using FFs, the MM energy of the system reads as
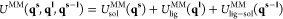
6in which *U*_sol_^MM^ is the energy of the solvent, which in this study consisted of CHCl_3_ molecules described by the MM model by Caldwell et al.;^90^*U*_lig_^MM^ corresponds to the energy of the ligand (γ-fluorohydrin), herein described using either GAFF or GAFF.MOD; *U*_lig–sol_^MM^ is the ligand–solvent (γ-fluorohydrin–CHCl_3_) interaction energy, a term that depends on the LJ parameters of GAFF (the same as those used by the traditional AMBER FF) and on the system partial charges; and **q**^**s**^, **q**^**l**^, and **q**^**s–l**^ are the degrees of freedom of the solvent, ligand, and interactions between them, respectively.

As an attempt to improve the accuracy of the pure MM model represented in eq 6, several studies^49,58−60^ have been conducted in which an ML model was employed to represent the ligand term, *U*_lig_^MM^(**q**^**l**^). Besides having the advantage of avoiding the parametrization of individual ligands, this hybrid model has led, in general, to higher accuracy in simulations. Because of the similarity of this hybrid scheme to the QM/MM model, it has come to be known as the NNP/MM model. The NNP/MM energy of a system in which only the ligand is included in the ML region and there are no covalent bonds between the ligand and the solvent reads as
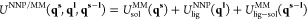
7Note that the only change in eq 7 relative to eq 6 is that the intramolecular representation of the ligand (γ-fluorohydrin) is now made by the ANI-2x NNP. Hence, since the ligand–solvent (γ-fluorohydrin–CHCl_3_) and solvent–solvent (CHCl_3_–CHCl_3_) interactions are still treated at the MM level, this hybrid model corresponds to a mechanical embedding scheme^57^ in which the partial charges of the ML region are kept fixed. As pointed out by Lahey and Rowley,^58^ FFs are parametrized in an internally consistent manner. Consequently, there is a chance that the MM parameters used to described the ligand–solvent nonbonded interactions are not optimal for the NNP/MM potential. The degree to which these nonoptimal nonbonded parameters may cause an imbalance between different parts of the model (in our case, between the MM ligand–solvent intermolecular interactions and the NNP ligand intramolecular interactions) is unknown *a priori* and must be investigated.

### 2.4 Molecular Dynamics Simulations

The gas-phase MD simulations were performed in the *NVT* ensemble using a Langevin integrator with a temperature of 298.15 K and a friction coefficient of 2 ps^–1^. These simulations ran for 100 ns with a time step of 1 fs. They were performed in triplicate for GAFF and GAFF.MOD, whereas for ANI-2x only one simulation per molecule was run for reasons of computational cost. For the simulations in chloroform, which used the same Langevin integrator settings as the gas-phase simulations, the chloroform box was created by adding CHCl_3_ molecules around the γ-fluorohydrins for 20 Å in the positive and negative *x*, *y*, and *z* directions. The solvated systems were then equilibrated in the *NPT* ensemble during 1 ns using the Monte Carlo barostat^91,92^ to fix the pressure at 1 bar. The LJ cutoff was set at a distance of 12 Å with a switching distance of 10 Å. Long-range electrostatic interactions were handled using the particle mesh Ewald (PME) method.^93,94^ The final *NVT* production runs were performed in duplicate (ANI-2x) or triplicate (GAFF and GAFF.MOD) during 100 ns. Snapshots of the trajectories were saved every picosecond. All MD simulations were run in OpenMM,^95^ and those that used ML models used the openmm-ml plugin that can be found at https://github.com/openmm/openmm-ml. The initial topology and coordinate files used as inputs to OpenMM and ParaMol were generated using LEaP.^96^

### 2.5 Populations of Conformers and Spin–Spin Coupling Constants

**Figure 2:**
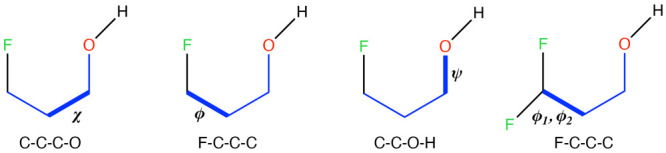
Dihedral angles used to identify the conformers of the γ-fluorohydrins.

The terminology used to identify the conformers of the γ-fluorohydrins follows that commonly employed to characterize the rotamers of protein side chains. Hence, the conformers arising from the rotation of the three threefold torsional barriers identified in Figure 2 are labeled according to the following definitions:^38,97,98^

- 0° ≤ χ, ϕ, ψ < 120° ⇒ g+
- 120° ≤ χ, ϕ, ψ < 240° ⇒ t
–120° ≤ χ, ϕ, ψ < 0° ⇒ g–

Using these labeling rules, the populations of the conformers obtained from MD simulations were estimated by clustering every individual frame of the final trajectories into the respective conformers. For the monofluoro derivatives, the conformers were identified by the sequence χϕ(ψ); for the difluoro derivatives, the conformers were identified by the sequences χϕ_1_ϕ_2_(ψ) or χϕ_2_ϕ_1_(ψ); and for trifluoro derivatives, the conformers were identified by the sequence χ(ψ). Furthermore, to have estimations of populations that are independent of the potential models being tested and that can thus be used as reference values, we calculated populations at various QM levels for the identified energetic minima of each γ-fluorohydrin. To do this, geometry optimizations and frequency calculations were carried out at the ωB97X/6-31G* and MP2/6-311++G(2d,p) levels of theory using Gaussian 09^99^ interfaced with ASE.^100^ Whenever required, solvent effects (CHCl_3_) were introduced through the polarizable continuum model (PCM). The Boltzmann populations of the conformers were then estimated from the calculated relative standard Gibbs free energies in the harmonic approximation.

The *J*-coupling constants were computed from geometries optimized at the ωB97X/6-311++G(2d,p)/PCM level of theory using the gauge-invariant atomic orbital (GIAO) method.^101−103^ In these calculations, the hybrid B97-2 functional^104^ and the pcJ-2 basis set,^105^ which exhibit good performance in the calculation of these NMR parameters,^38,106,107^ were used. Again, solvent effects were included through the PCM model. The calculated *J*-coupling constants were finally averaged over all conformers according to their relative populations in chloroform at 298.15 K using the following equation:
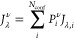
8in which λ identifies the *J*-coupling constant being considered and ν refers to the QM level or molecular model from which the populations are estimated.

## 3 Results and Discussion

### 3.1 nMC-MC Acceptance Rates

**Figure 3:**
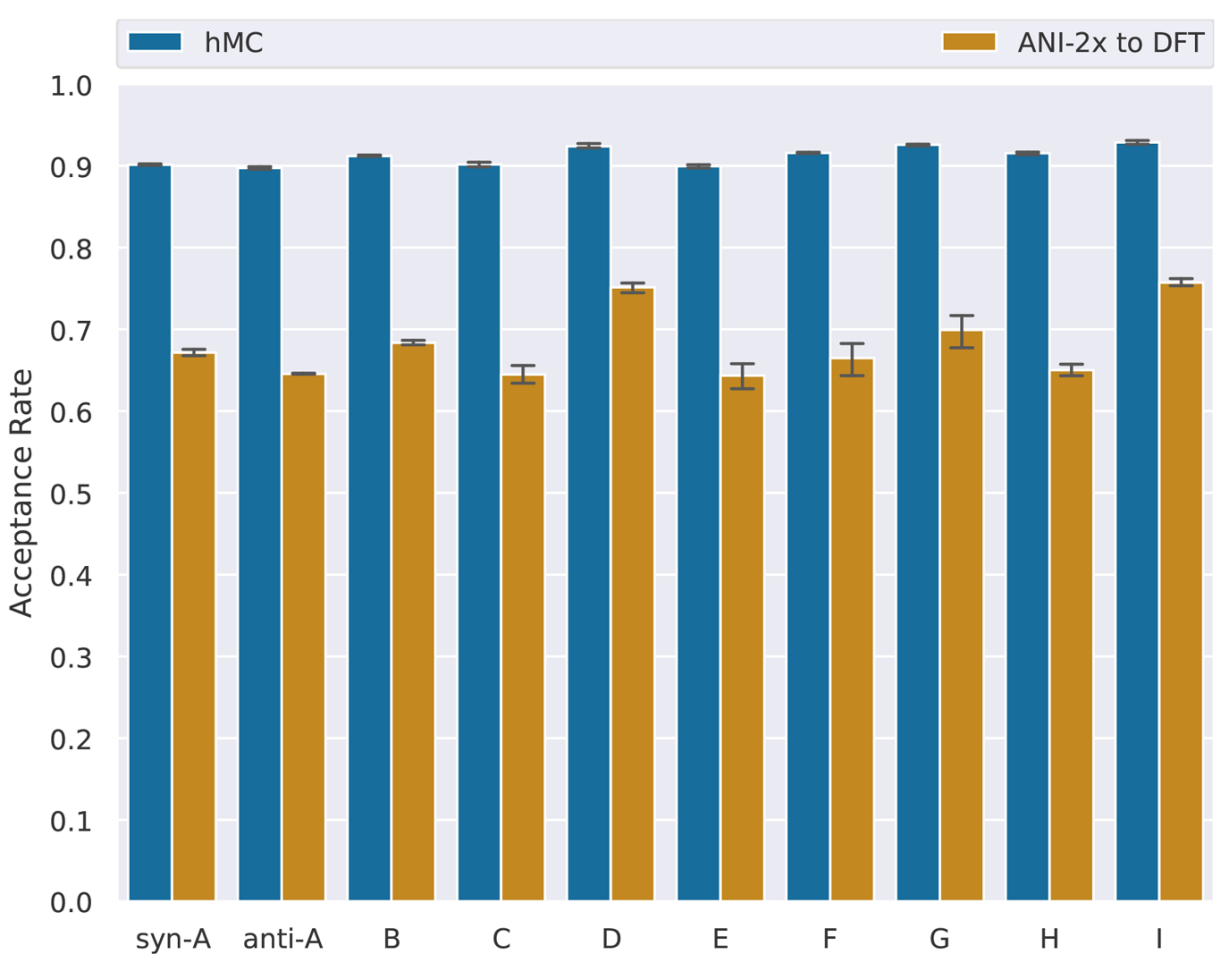
Acceptance rates obtained in the nMC-MC simulations for each γ-fluorohydrin. Only the three samplers that gave the lowest acceptance rates were included in the calculation of the mean and standard deviation of each bar.

We first present the results obtained in the nMC-MC simulations. The nMC-MC simulations aimed at generating ensembles of configurations representative of the ωB97X/6-31G* ensemble by using ANI-2x as the approximate potential. The acceptance rates that the nMC-MC simulations produce give important information about the ANI-2x performance in the gas phase.^21^ On the one hand, the hMC acceptance rate measures the stability of the short MD runs performed in the nMC-MC algorithm, and it is positively correlated with energy conservation during the MD run. Since we obtained hMC acceptance rates of ≥90% for all molecules in the test set (Figure 3), we conclude that ANI-2x is a viable model to use in MD simulations. These high hMC acceptance rates are comparable in magnitude to those we obtained in a previous study of molecules of similar size modeled using GAFF-like FFs.^21^ On the other hand, the ANI-2x to DFT acceptance rate measures the similarity between the ANI-2x potential and the ωB97X/6-31G* level of theory. We obtained acceptance rates of >60% in the ANI-2x to DFT step (Figure 3). Compared to the results of the previous study,^21^ these are very high acceptance rates, indicating excellent agreement between ANI-2x and ωB97X/6-31G*.

### 3.2 Energetic and Geometric Agreement in the Gas Phase

Comparing the performances of different ligand models in the absence of experimental data is not straightforward because of the lack of an absolute reference. FFs and ML potentials, however, are often fitted to reproduce gas-phase energies and geometries at specific QM levels of theory. This is the case for GAFF, which was fitted to reproduce experimental, MP2/6-31G* (equilibrium bonds and angles), and MP4/6-311G(d,p)//MP2/6-31G* (dihedral parameters) data,^55^ and for GAFF.MOD and ANI-2x, which were fitted to reproduce ωB97X/6-31G* data.^39^ Therefore, the natural way of benchmarking the accuracy of molecular models in the gas phase is to compare their performance to that of the QM level against which they were fitted. Here we present and discuss our gas-phase results by comparing the performances of GAFF, GAFF.MOD, and ANI-2x to reference QM data.

To assess the energetic agreement between the different molecular models and a given QM reference, we calculated relative energy differences using the following equation:^24^
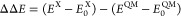
9in which X denotes the molecular model used (GAFF, GAFF.MOD, or ANI-2x), and the 0 subscript identifies the conformer with the lowest QM energy for the given molecule. Ideally, a model should give ΔΔ*E* values close to 0 kJ mol^–1^, indicating good agreement with the QM level. Broader distributions indicate larger deviations with respect to the QM level. A negative ΔΔ*E* value indicates that relative energy calculated by the model is underestimated compared to the QM relative energy, whereas a positive ΔΔ*E* value indicates that the relative energy calculated by the model is overestimated compared to the QM relative energy.

**Figure 4:**
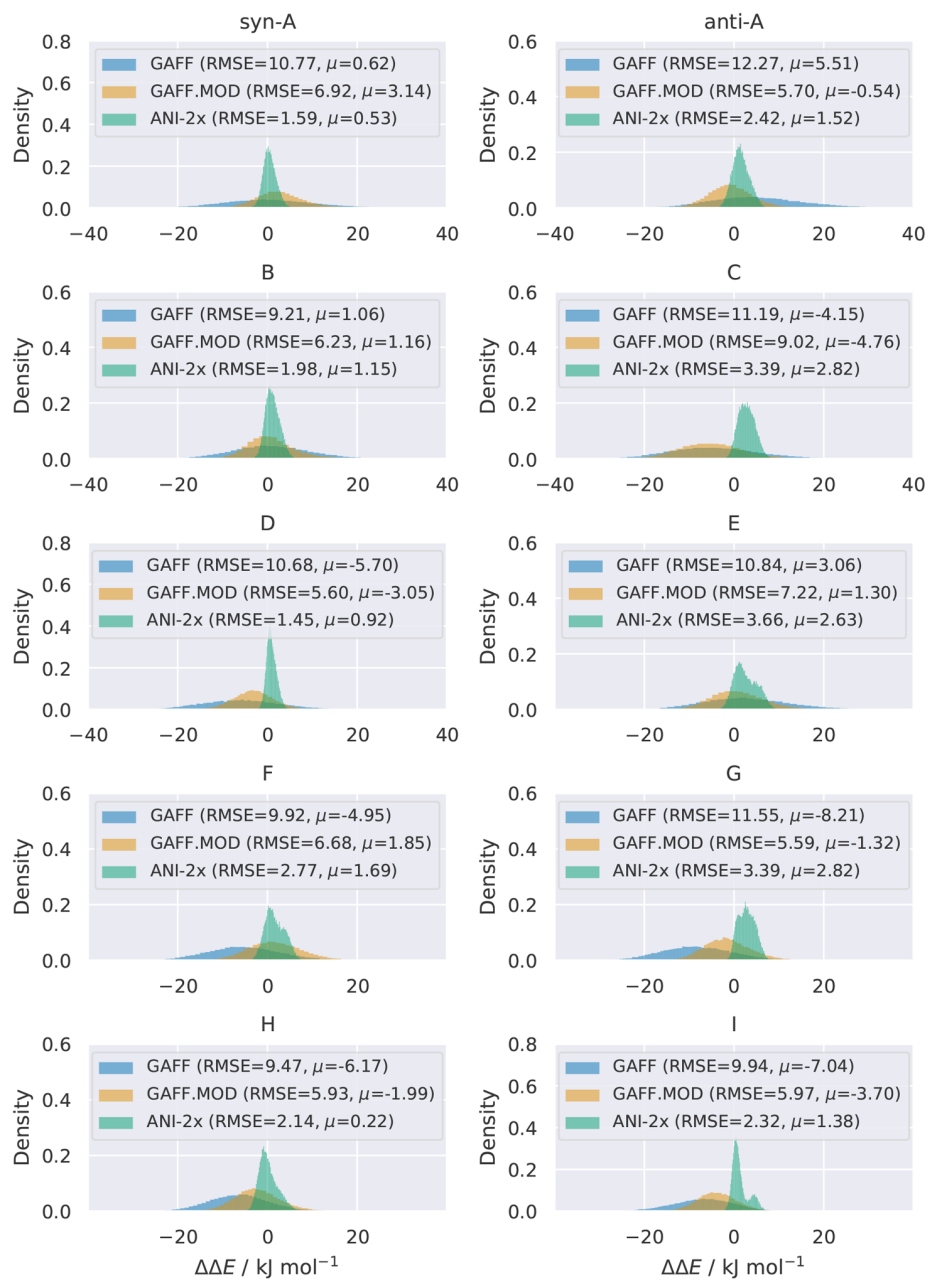
Distributions of the relative energy differences (ΔΔ*E*) for GAFF, GAFF.MOD, and ANI-2x with respect to the ωB97X/6-31G* level of theory. The RMSE and the mean (μ) are indicated for each distribution. Each testing data set was composed of 3 × 10^4^ structures extracted from the nMC-MC simulations. The molecular structures used as a reference were removed from the histograms. Note that the color scaling is different in each plot.

For each γ-fluorohydrin, we calculated the relative energy differences for the three molecular models relative to ωB97X/6-31G* using 3 × 10^4^ structures extracted from the nMC-MC simulations. The distributions of the relative energy differences show an unequivocal trend: the performance of the models tested decreases as ANI-2x > GAFF.MOD > GAFF (Figure 4). This presents evidence that ANI-2x excels in reproducing the level of theory it has been trained to reproduce, with root-mean-square errors (RMSEs) for all molecules below the chemical accuracy of 4.184 kJ mol^–1^ (1 kcal mol^–1^). GAFF.MOD, the optimally tuned FF optimized using ωB97X/6-31G* data, underperforms relative to ANI-2x but still shows notable improvements relative to the original GAFF. Importantly, the GAFF.MOD distributions invariably show increased precision (narrower distributions) and, for most molecules, increased accuracy (mean of the distributions closer to zero) than GAFF, confirming that reparametrization improved the FF agreement with ωB97X/6-31G*.

Several confounding factors may have contributed to preventing GAFF.MOD from achieving the same level of accuracy as ANI-2x. First, since GAFF.MOD is a GAFF-like model, it is constrained by the FF functional form, which may not have been ideal to energetically represent some configurations sampled in the ωB97X/6-31G* ensemble. Second, to derive GAFF.MOD, only the GAFF bonded parameters were optimized, leaving the nonbonded parameters untouched. It is well-known that ωB97X lacks dispersion interactions since the semilocal correlation functionals cannot capture long-range correlation effects.^51,108−111^ This physical artifact may not have been entirely captured by only optimizing the bonded part of GAFF, as dispersion physics is modeled by the LJ 12–6 potential. Third, as the objective function (eq 3) included a regularization term that depends on the initial FF parameters, the solutions of the optimization problem were dependent on this initial guess (in the present work, the GAFF parameters). We cannot exclude the possibility of obtaining higher-quality FF parameters if another initial guess were used, though this also poses the nontrivial problem of determining alternative initial guesses. Fourth, the completeness of the training data set used in the reparametrization procedure may also have impacted the quality of the optimized FF parameters. The nMC-MC simulations, however, generated representative ωB97X/6-31G* ensembles. We thus believe the completeness of the training data set did not have a significant impact on the quality of the reparametrization procedure. These four issues could be addressed by using either more advanced FF functional forms or by changing the nature of the optimization procedure, work which is outside the scope of the current study.

**Figure 5:**
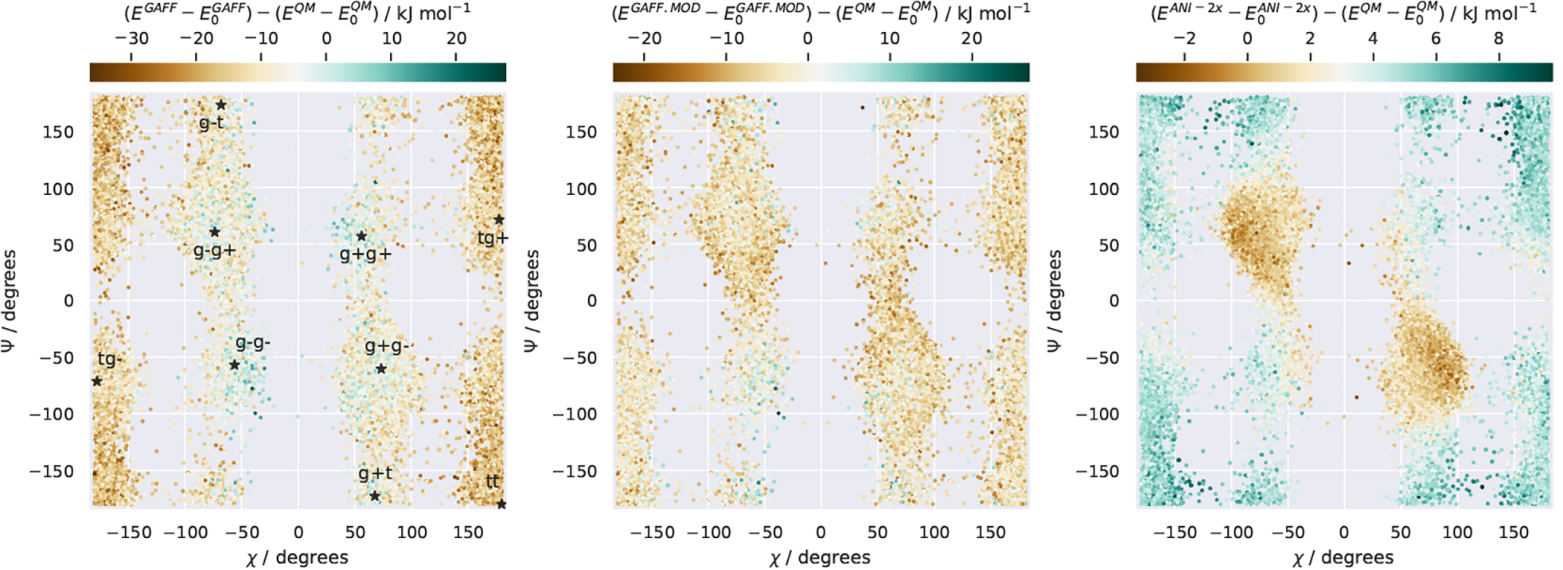
Distributions of the χ and ψ dihedral angles (see definitions in Figure 2) of molecule I for configurations sampled using three nMC-MC simulations. The color of each point gives the relative energy difference (ΔΔ*E*) between the model (GAFF, left; GAFF.MOD, middle; ANI-2x, right) and ωB97X/6-31G*. The black stars locate the QM minima calculated using ωB97X/6-31G*.

Two types of errors are present when molecular models are used to perform energy measurements: random errors and systematic errors. Both originate from functional form constraints and/or inadequate model parameters. Random errors are related to the precision of the molecular model and are normally distributed around the true value. Systematic errors are related to the accuracy of the molecular model and cause the mean of the error distribution to deviate from the true value and/or the error distribution to be non-Gaussian. The distributions of the relative energy differences (Figure 4) show that GAFF has a bias toward negative relative energy differences for most molecules (C, D, F, G, H, and I). On the other hand, a bias toward positive relative energy differences is seen for molecules *anti*-A and E, whereas for molecules *syn*-A and B random errors dominate. The systematic errors for the GAFF model are mostly offset errors, as they manifest themselves as deviations from the true value (the distributions remain approximately Gaussian). Furthermore, the GAFF.MOD distributions tend to have a mean closer to the true value (smaller offset errors) than GAFF, while also showing smaller random errors. Finally, although the magnitude of the random errors of ANI-2x are small, they present significant systematic errors that lead to very pronounced non-normally distributed relative energy differences. The distribution of molecule I is of particular concern due to its bimodal shape. This bimodal shape occurs because conformations g–(g+) and g+(g−) of molecule I have a systematic bias toward negative relative energy differences, whereas the remainder conformations have a systematic bias toward positive relative energy differences (Figure 5). As we shall see later in the discussion, systematic errors with conformation-dependent signs and/or magnitudes scale the relative energy between conformers, consequently changing their relative populations, which is an undesirable situation in the context of conformational sampling and optimization.

**Figure 6:**
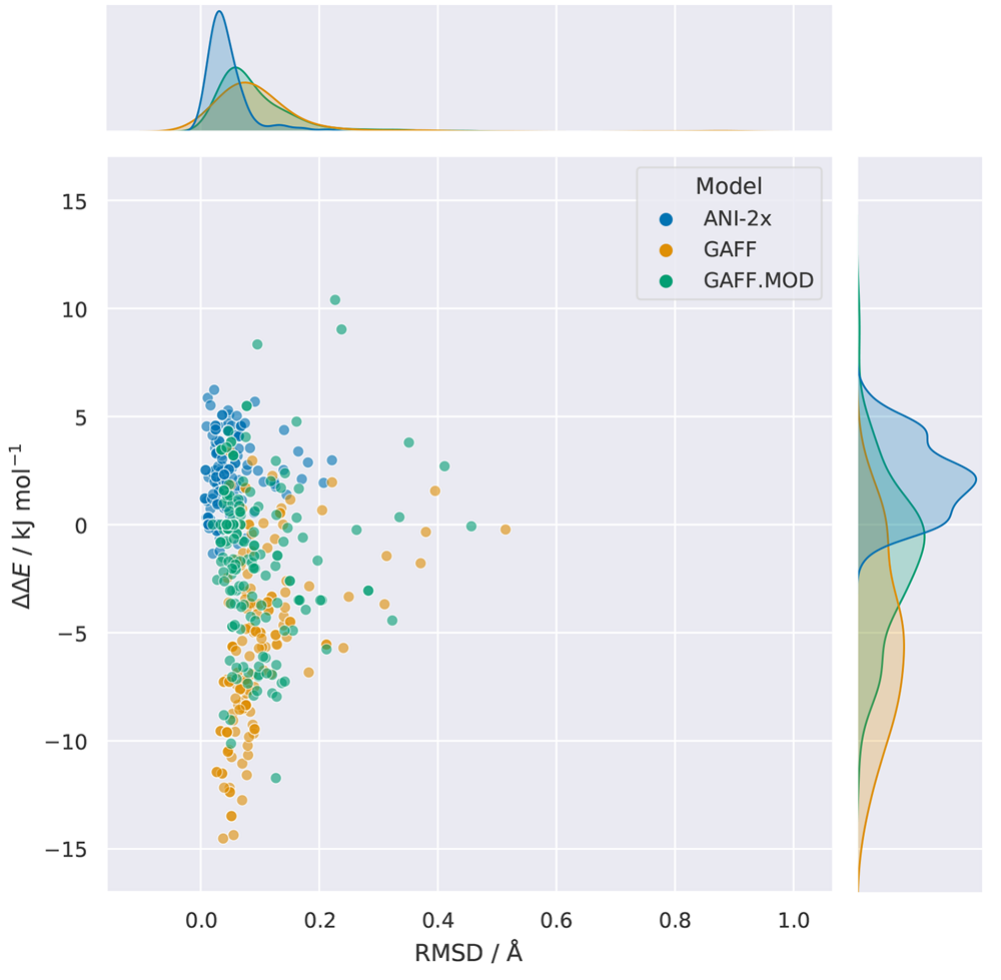
Scatter plots of the relative conformer energies (ΔΔ*E*) vs the RMSD of atomic positions. Each point was obtained by performing a geometry optimization using GAFF, GAFF.MOD, or ANI-2x, starting from all QM minima within 12.552 kJ mol^–1^ (3 kcal mol^–1^) of the global minimum. The QM reference is the ωB97X/6-31G* level of theory.

Next, we will discuss the performance of the models in reproducing the energies and geometries of the QM minima. An ideal model should yield optimized geometries similar to QM, and the relative energies of those minima should agree between the models and QM.^24^ To assess performance in these two categories, we performed geometry optimizations using GAFF, GAFF.MOD, and ANI-2x starting from all QM minima within 12.552 kJ mol^–1^ (3 kcal mol^–1^) of the global minimum. The GAFF and GAFF.MOD geometry optimizations were performed with the L-BFGS algorithm of OpenMM (tolerance = 0.001), and the ANI-2x geometry optimizations were performed using the BFGS algorithm of ASE (fmax = 0.001). The RMSDs of the relative energy differences and the average RMSD of the atomic positions are shown in Table 1. The results obtained agree with the findings presented so far, indicating that ANI-2x is the model that best reproduces the energies and geometries of the ωB97X/6-31G* minima, followed by GAFF.MOD and then by GAFF (Figure 6). Interestingly, we observe that ANI-2x tends to predict positive relative energy differences, meaning that the relative energies between the local and global minima tend to be overestimated. This observation is cause for concern, as it indicates that the ANI-2x global minima tend to be systematically overstabilized. Furthermore, GAFF tends to underestimate the relative energy differences, whereas GAFF.MOD errors were mostly random, though still presenting a non-negligible tendency to underestimate the relative energies of some minima relative to QM. As expected, the inverse trend is observed when the QM reference is MP2/6-311++G(2d,p), indicating that GAFF is the model that gives the best energetic agreement with this QM level, followed by GAFF.MOD and ANI-2x (Figure S1). Moreover, the average RMSD of the atomic positions is lower for GAFF.MOD and ANI-2x than for GAFF, though these results are heavily influenced by outliers. Removing all points with an RMSD greater than 0.3 Å leads to equal trends for the energetic and geometric agreement, as expected.

**Table 1:** RMSDs of the Relative Energy Differences (ΔΔ*E*, in kJ mol^–1^) and Average RMSDs of Atomic Positions (in Å) for the Scatters Depicted in Figure 6^a^

	ΔΔ*E*		atomic positions	
model	MP2	ωB97X	MP2	ωB97X
GAFF	3.36	7.45	0.087	0.110
GAFF.MOD	4.26	4.05	0.079	0.095
ANI-2x	5.12	2.80	0.080	0.046

a The molecular structures used as a reference were excluded from the calculation of the RMSDs of the relative energy differences. The QM references are MP2/6-311++G(2d,p) (MP2) and ωB97X/6-31G* (ωB97X).

### 3.3 Sampling Accuracy in the Gas Phase

**Figure 7:**
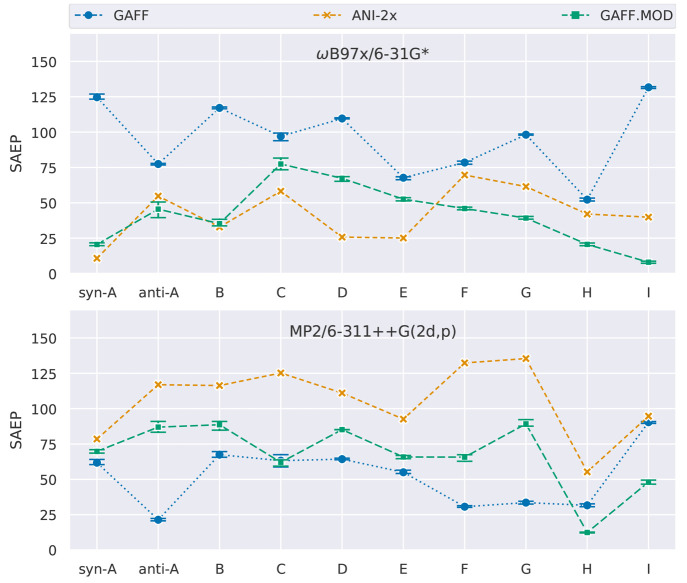
Sum of the absolute error of the populations (SAEP), calculated as the absolute difference between the populations predicted by the models (GAFF, GAFF.MOD, and ANI-2x) and the QM level. The QM references are (top) ωB97X/6-31G* and (bottom) MP2/6-311++G(2d,p).

We also assessed the sampling accuracy of each model in the gas phase by comparing the populations of the γ-fluorohydrins, as predicted by MD, to their Boltzmann populations calculated at ωB97X/6-31G* and MP2/6-311++G(2d,p). To estimate the QM populations, the electronic energies were converted into Gibbs free energies in the harmonic approximation using standard thermodynamic corrections obtained from frequency calculations.^38^ This approach introduces two approximations: it considers noninteracting particles and assumes that the first and higher electronic excited states are entirely inaccessible.^112^ While the latter assumption should not pose a problem for the set of γ-fluorohydrins considered in this study, the former may introduce some error, depending on how much the systems deviate from ideal behavior. Whenever possible, the QM populations should be estimated by performing MD or MC simulations. However, owing to the prohibitive computational cost of *ab initio* simulations, we believe that our approach is sufficiently reasonable to warrant investigation. The metric we used to evaluate the sampling accuracy was the sum of the absolute error of the populations (SAEP), calculated as the absolute difference between the populations predicted by the model X and the QM level, such that
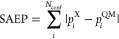
10in which *p*_*i*_ is the population of the *i*th conformer and *N*_conf_ denotes the total number of conformers. When ωB97X/6-31G* is used as the reference, it is not possible to determine the model that predicts the best sampling accuracy because mixed results were obtained (Figure 7, top panel). For example, ANI-2x exhibits significantly higher sampling accuracy than GAFF.MOD for molecules C, D, and E, but GAFF.MOD exhibits significantly higher sampling accuracy than ANI-2x for molecules F, G, H, and I. There are also some molecules (*syn*-A, *anti*-A, and B) for which GAFF.MOD and ANI-2x perform similarly. GAFF, however, stands out as the model that worst reproduces the populations at ωB97X/6-31G*. This trend is inverted when MP2/6-311++G(2d,p) is used as the reference (Figure 7, bottom panel), indicating that there is a significant disagreement between the predictions of MP2/6-311++G(2d,p) and ωB97X/6-31G*. This is, however, expected behavior: while ANI-2x was trained to reproduce the ωB97X/6-31G* level of theory, GAFF was derived using experimental, MP2, and MP4 data.^55^ Hence, it is natural that GAFF is the model that overall best reproduces the populations at MP2/6-311++G(2d,p). Interestingly, GAFF.MOD FFs still perform reasonably well when MP2/6-311++G(2d,p) is the reference, and they actually outperform GAFF for the trifluoro derivatives H and I because the populations for these molecules are similar in the two QM references. The good performance of the GAFF.MOD FFs is attributed to the fact that their parameters, while optimized to reproduce ωB97X/6-31G*, retain some “memory” of the original GAFF parameters due to the regularization applied during the optimization procedure.

**Figure 8:**
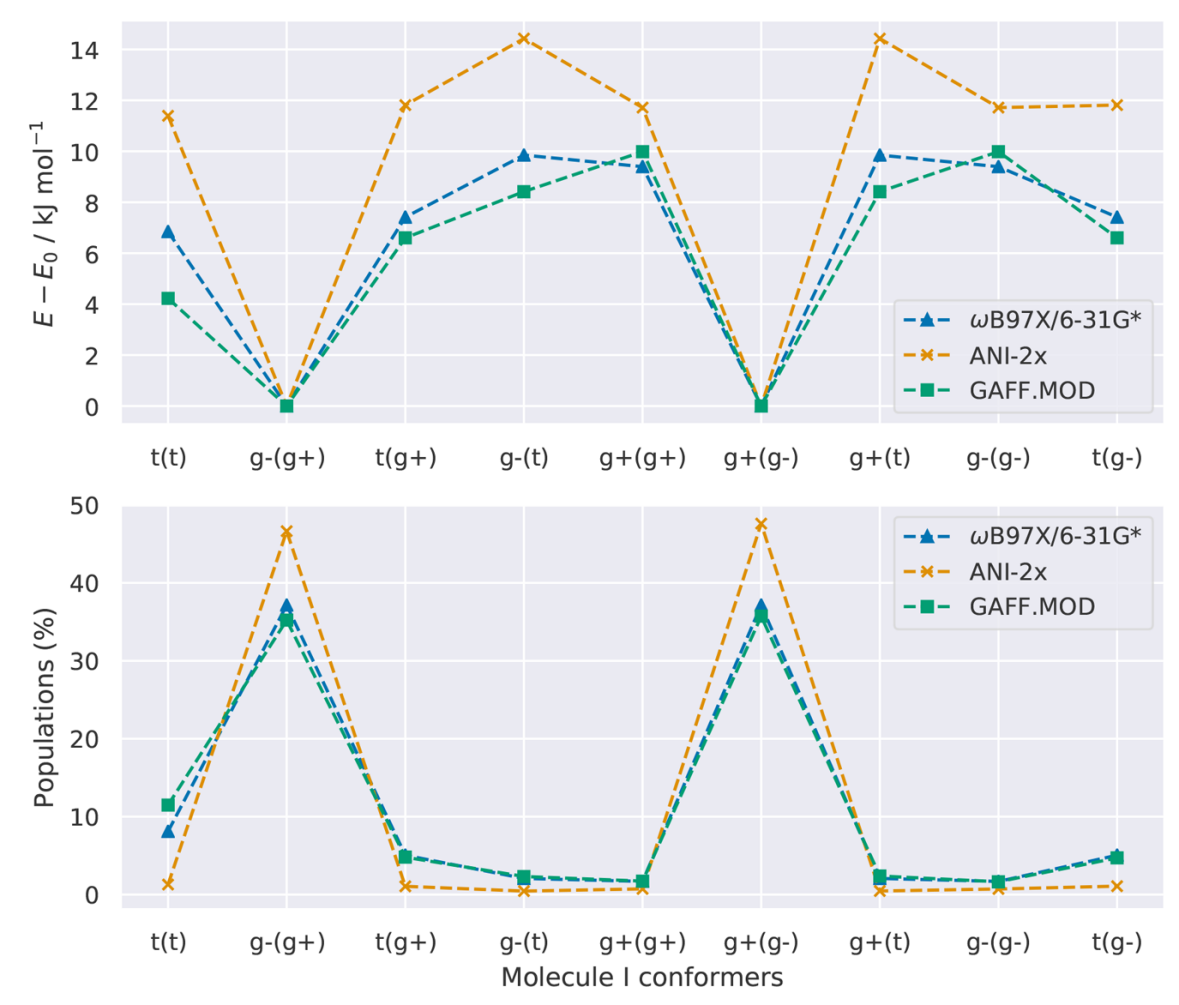
(top) Relative energies of the conformers of molecule I (optimized geometries), calculated using ωB97X/6-31G*, ANI-2x, and GAFF.MOD. (bottom) Populations of each conformer of molecule I as predicted by ωB97X/6-31G*, ANI-2x, and GAFF.MOD.

Two reasons may have contributed to GAFF.MOD predicting a significantly lower SAEP for some molecules (F, G, H, and I) than ANI-2x, even though the GAFF.MOD energy landscape agreement with ωB97X/6-31G* is lower than that of ANI-2x (Figure 4). The first reason is energetic: energetic errors that affect the relative energies of conformers change their relative populations. For example, if the relative energy between two conformers increases, the conformer with lower energy becomes more populated. The second reason is entropic: populations depend not only on conformational energies but also on conformational entropies.^113^ Broader wells are associated with more configurations than narrower wells, being more entropically favorable. For ANI-2x, we observe conformation-dependent directions of bias (Figures 5), a tendency to overstabilize the global minima (Figure 8), and a tendency to underestimate the conformational entropy^114^ (Figure S5). Hence, it was a combination of energetic and entropic factors that, for some molecules, caused the sampling accuracy of GAFF.MOD to be closer to ωB97X/6-31G* than that of ANI-2x.

In summary, the results obtained in the gas-phase benchmark demonstrate that models tend to behave similarly to the QM levels to which they have been fitted. ANI-2x exhibits levels of accuracy that class I FFs (GAFF and GAFF.MOD) cannot achieve when using ωB97X/6-31G* as the reference (Figure 4). Interestingly, the high accuracy of ANI-2x in reproducing the ωB97X/6-31G* energy landscape does not always translate into high sampling accuracy (Figure 7). For some molecules, ANI-2x exhibits systematic errors that cause significant overestimation of the relative energies between the local and global minima, leading to an overpopulation of the lower-energy conformers (Figures 6 and 8). This observation raises concerns about whether to use ANI-2x over a conventional FF to sample the conformational landscape, as the computational cost of ANI-2x is up to 100 times greater than that of a class I FF. These concerns are further aggravated by realizing that ANI-2x performs the worst in reproducing MP2/6-311++G(2d,p) (Figure 7), which in principle is more accurate than ωB97X/6-31G*. This difference suggests that ωB97X/6-31G* and MP2/6-311++G(2d,p) predict different physical behavior for the γ-fluorohydrins considered in this study. In the next sections, we evaluate the performance of the models in chloroform solution and then proceed to assess which QM level best reproduces experimentally determined *J*-coupling constants.

### 3.4 Sampling Accuracy in Chloroform Solution

**Figure 9:**
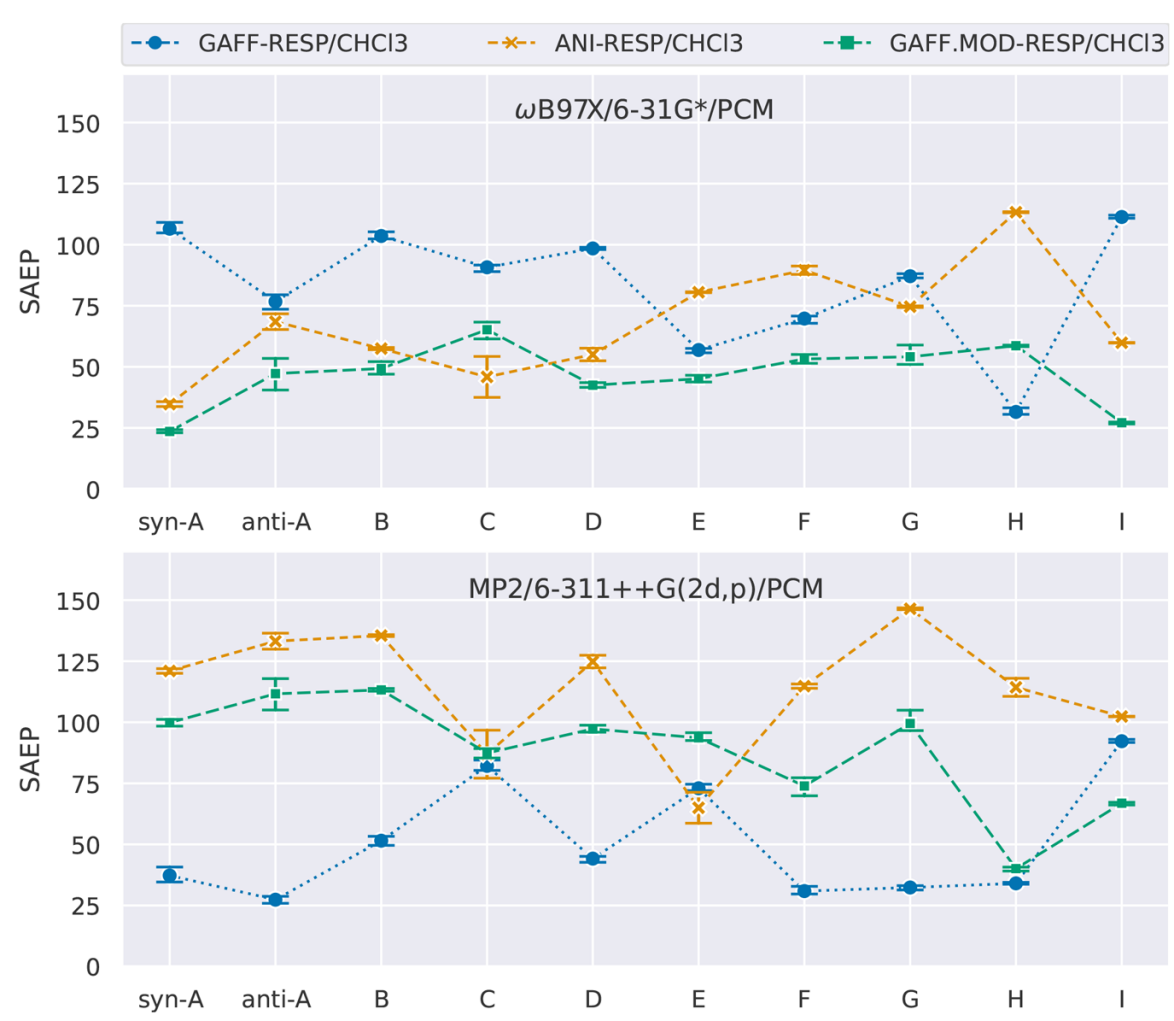
Sum of the absolute error of the populations (SAEP), calculated as the absolute difference between the populations predicted by the models (GAFF-RESP/CHCl_3_, GAFF.MOD-RESP/CHCl_3_, and ANI-2x-RESP/CHCl_3_) and the QM level. The QM references are (top) ωB97X/6-31G*/PCM and (bottom) MP2/6-311++G(2d,p)/PCM.

We begin the discussion of the results obtained in chloroform solution by assessing the sampling accuracy of each model. To do this, we follow the procedure presented in the previous section and compare the populations of the γ-fluorohydrins, as predicted by MD, to their Boltzmann populations calculated at ωB97X/6-31G*/PCM and MP2/6-311++G(2d,p)/PCM. This approach assumes that these QM levels and solvent model represent the standard against which we compare. GAFF.MOD-RESP/CHCl_3_ is the model that overall predicts better sampling accuracy when ωB97X/6-31G*/PCM is used as the reference (Figure 9). Specifically, GAFF.MOD-RESP/CHCl_3_ best reproduces the populations of molecules *syn*-A, *anti*-A, B, D, E, F, G, and I, whereas the populations of molecules C and H are best reproduced by ANI-2x-RESP/CHCl_3_ and GAFF-RESP/CHCl_3_, respectively. Compared to the gas-phase results (Figure 7), in which ANI-2x and GAFF.MOD performed similarly, GAFF.MOD seems to be better suited for condensed-phase simulations. Since in the ANI-2x-RESP/CHCl_3_ simulations the LJ 12–6 parameters were taken from GAFF, these results suggest that there is an imbalance between the ligand–solvent intermolecular and ligand intramolecular interactions. Hence, for some systems, the practice^49,58−60^ of directly combining LJ 12–6 parameters with NNPs may decrease the NNP performance in the condensed phase. This observation strongly indicates that sets of LJ 12–6 parameters consistent with ANI-2x should be developed in the future so that the NNP gas-phase accuracy does not decrease in the condensed phase. The optimally tuned GAFF.MOD FFs, on the other hand, despite having been optimized to reproduce ωB97X/6-31G*, present a better balance between the ligand–solvent intermolecular and ligand intramolecular interactions. The GAFF.MOD-RESP/CHCl_3_ are models with greater internal consistency than ANI-2x-RESP/CHCl_3_ because their parameters are closer to those of GAFF owing to the regularization applied during the optimization procedure.

**Figure 10:**
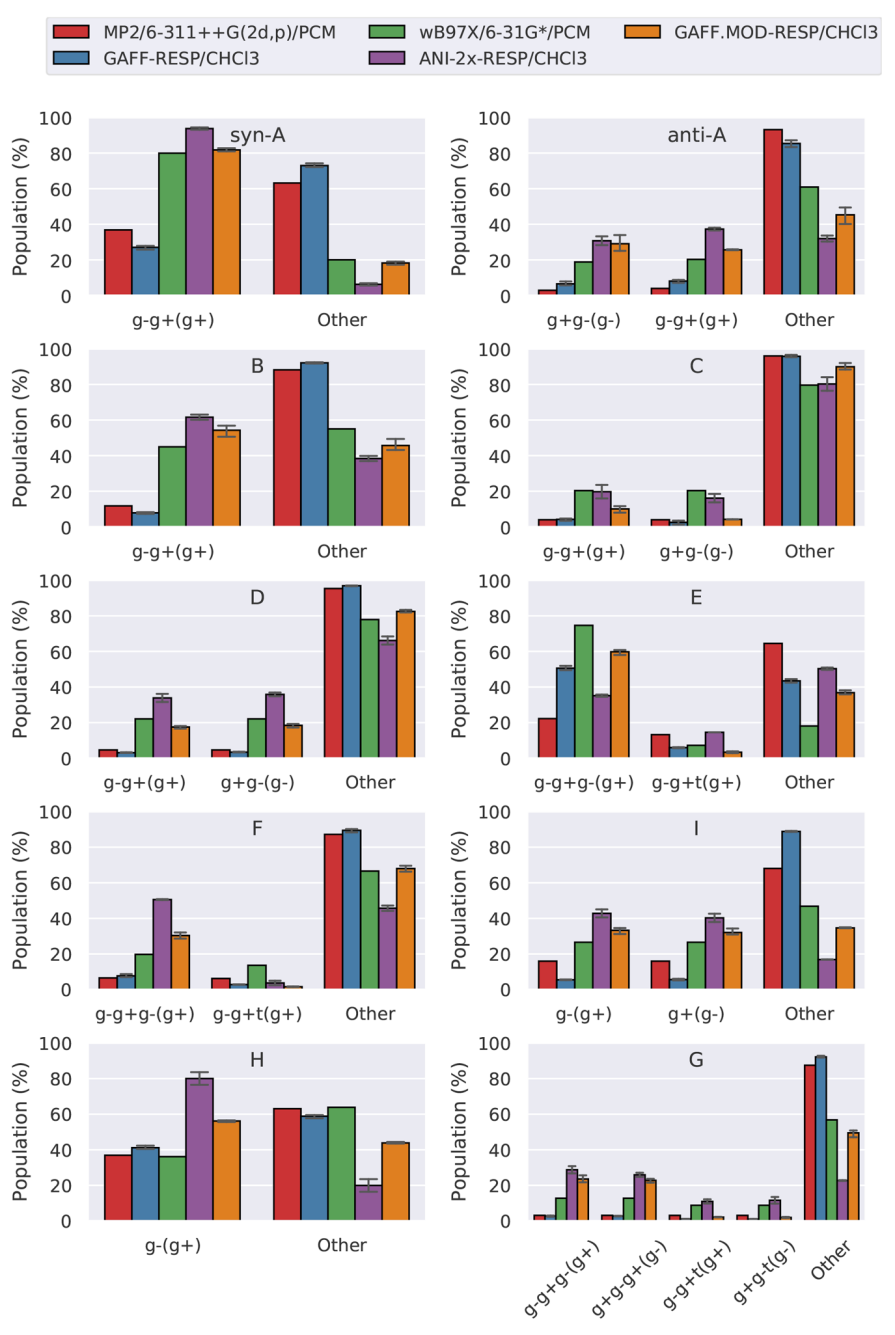
Populations in chloroform solution of the conformers with IMHBs.

When the reference is MP2/6-311++G(2d,p)/PCM, GAFF-RESP/CHCl_3_ is the model that overall predicts better sampling accuracy. Exceptions occur for molecule C, for which all models perform similarly; molecule E, for which ANI-2x-RESP/CHCl_3_ and GAFF-RESP/CHCl_3_ perform similarly; and molecule I, for which GAFF.MOD-RESP/CHCl_3_ performs the best. Following the previously presented discussion for the gas-phase benchmark, this is expected behavior since GAFF was fitted to a QM level similar to MP2/6-311++G(2d,p). Again, the differences in results obtained when using different QM references indicate that the QM references predict different physical behavior. To understand how this relates to the sampled conformers, we determined the populations of the conformers with IMHBs for each model and QM reference (Figure 10). From these results, we see that ωB97X/6-31G*/PCM tends to overestimate the populations of the conformers with IMHBs relative to MP2/6-311++G(2d,p)/PCM. This overestimation trend is even more pronounced for the ANI-2x-RESP/CHCl_3_ model. As has been shown previously, ANI-2x tends to overestimate the relative energies of local minima relative to the global minima (Figures 6 and 8). Since for this set of γ-fluorohydrins the global minima are mostly the conformers with IMHBs, it follows that the overpopulation of these conformers relative to ωB97X/6-31G*/PCM is a consequence of this energetic error of the ANI-2x model. Similar results were obtained in the gas phase (Figure S2), indicating that this problem cannot be entirely attributed to the imbalance of the hybrid NNP/MM scheme here employed, as part of it is a consequence of the errors intrinsic to ANI-2x.

It is well-known that ωB97X/6-31G* lacks dispersion interactions, and thus, it is expected that ANI-2x also suffers from this physical artifact. Incidentally, a previous study has shown that the absence of dispersion interactions in ANI-2x negatively affects the modeling of bulk water and peptides, as it led to stronger-than-expected hydrogen bonds (HBs).^51^ For our simulations in chloroform solution, we also observe this phenomenon, as ANI-2x-RESP/CHCl_3_ and ωB97X/6-31G*/PCM predicted shorter HBs than GAFF-RESP/CHCl_3_ and MP2/6-311++G(2d,p)/PCM (Figure S3). The results obtained could potentially be improved by including a dispersion correction, such as D3.^115^ However, it is unclear whether this correction would mitigate the systematic errors in relative energies observed for ANI-2x. Alternatively, for molecules only containing elements H, C, N, and O, the ANI-1cxx NNP could be applied, as in principle it properly captures dispersion interactions. It would also be possible to run MD simulations in which both the solute and solvent were described at the NNP level. However, our attempts to simulate bulk chloroform using ANI-2x led to radial distribution functions (RDFs) that indicate an overstructuring tendency relative to the experimental RDFs (Figure S4). Again, this overstructuring is likely caused by the lack of dispersion interactions, which causes permanent dipole–dipole interactions to dominate. Note that ANI-2x was not trained to reproduce bulk chloroform. Nevertheless, ANI-2x was trained to reproduce bulk water, and the overstructuring tendency is still observed.^51^ Additionally, the short-range nature of ANI-2x requires bulk NNP simulations to use high-pressure values (ca. 1540 bar for our bulk chloroform simulation) for the barostat so that bulk densities comparable with the experiment are obtained. The absence of long-range electrostatic interactions in ANI-2x naturally suggests the use of hybrid NNP/MM schemes, as long-range interactions are easily computed at the MM level. Unfortunately, NNP/MM hybrid models pose additional and still unsolved problems because, to obtain high levels of accuracy, they require sets of nonbonded parameters consistent with the NNP. In the absence of these parameters, optimally tuned FFs seem to be the most viable alternative to use for cases in which the original FF performs poorly, as for our test set optimally tuned FFs led to higher sampling accuracy than ANI-2x, with the advantage of having a much lower computational cost.

### 3.5 NMR *J*-Couplings

In the last section of the discussion, we present the results obtained for the *J*-coupling constants (^h1^*J*_OH···F_). We resort to the experimental NMR data available in chloroform solution^38^ to determine which molecular model or QM level gives the populations that best reproduce the true behavior of the molecules considered in this study. To estimate the theoretical *J*-couplings, we used eq 8 to average the calculated ^h1^*J*_OH···F_ values at B97-2/pcJ-2/PCM//ωB97X/6-311++G(2d,p)/PCM (NMR calculation//geometry optimization) over the conformer populations predicted by each model or QM level.

By analyzing the results given in Table 2, we conclude that MP2/6-311++G(2d,p)/PCM is the QM level of theory that best reproduces the experimental *J*-couplings, as it gave the highest squared Pearson correlation coefficient (*R*^2^ = 0.96) and lowest RMSE (0.41 Hz). ωB97X/6-31G*/PCM gave the second-best *R*^2^ value (0.87), indicating a strong correlation between theoretical and experimental data. The *R*^2^ values, however, do not reflect systematic errors, which are high for this DFT functional, as can be seen from its RMSE value (4.81 Hz). Concerning the molecular models, GAFF-RESP/CHCl_3_ gave a lower RMSE (1.27 Hz) than GAFF.MOD-RESP/CHCl_3_ (4.77 Hz), though with a smaller *R*^2^ value (0.68 vs 0.75). As low RMSE values indicate both high precision and accuracy, we consider RMSE to be a better metric than *R*^2^ to measure the agreement with the experiment. Under this assumption, GAFF-RESP/CHCl_3_ is the model that best reproduces the experimental data, which is justified by its similarity to the MP2 level. Finally, ANI-2x-RESP/CHCl_3_ exhibits the greatest disagreement with the experiment, presenting the lowest *R*^2^ (0.58) and highest RMSE (6.83 Hz) values. We believe that this occurs because as well as suffering from the same physical artifacts as ωB97X/6-31G* (viz., lack of dispersion interactions and stronger-than-expected hydrogen bonds), ANI-2x further systematically overstabilizes global minima, impacting the populations entering in eq 8.

**Table 2:** Experimental and Computed *J*-Couplings (^h1^*J*_OH···F_) Obtained in CDCl_3_

molecule	exptl^a^	MP2^b^	ωB97X^c^	GAFF^d^	GAFF.MOD^e^	ANI-2x^f^
*syn*-A	6.6	–7.6	–16.5	–5.6 ± 0.2	–16.1 ± 0.2	–19.6 ± 0.2
*anti*-A	1.9	–1.1	–7.4	–2.6 ± 0.4	–9.9 ± 0.6	–13.0 ± 0.4
B	2.2	–2.2	–9.2	–1.6 ± 0.1	–10.8 ± 0.2	–13.6 ± 0.1
C	1.7	–1.2	–6.6	–1.1 ± 0.2	–2.2 ± 0.3	–5.8 ± 0.3
D	1.4	–1.3	–6.7	–0.85 ± 0.03	–5.4 ± 0.1	–10.7 ± 0.3
E	3.5	–3.2	–11.3	–7.7 ± 0.2	–9.0 ± 0.2	–5.3 ± 0.1
	1.4	–1.7	–1.0	–0.7 ± 0.1	–0.6 ± 0.4	–3.0 ± 0.3
F	0.6	–0.6	–1.8	–0.25 ± 0.03	–0.4 ± 0.1	–0.3 ± 0.2
	0.6	–0.7	–2.8	–0.9 ± 0.1	–4.1 ± 0.2	–7.9 ± 0.1
G	0.4	–0.1	–1.1	–0.06 ± 0.05	–2.2 ± 0.2	–2.7 ± 0.3
	0.4	–0.3	–2.0	–0.11 ± 0.02	–2.3 ± 0.1	–3.624 ± 0.004
H	0.7 (q)^g^	–0.8	–0.8	–0.92 ± 0.03	–1.46 ± 0.01	–2.68 ± 0.04
I	0.3 (q)^g^	–0.4	–1.0	0.39 ± 0.01	–1.29 ± 0.01	–1.765 ± 0.002
*R*^2^		0.96	0.87	0.68	0.75	0.58
RMSE		0.41	4.81	1.27	4.77	6.83

a Sign not determined.

b MP2/6-311++G(2d,p)/PCM.

c ωB97X/6-31G*/PCM.

d GAFF-RESP/CHCl_3_.

e GAFF.MOD-RESP/CHCl_3_.

f ANI-2x-RESP/CHCl_3_.

g Quartet.

Three possible sources of error can impact the accuracy of the calculated *J*-coupling constants: the error in the populations, which is directly related to the model or QM level of theory used to estimate them; the error of the QM method used to calculate the *J*-coupling values for each conformer; and the error related to the implicit solvent model. Even though these errors are not easily quantified, we attempted to determine the error associated with the QM method employed to calculate the *J*-couplings by determining the ^h1^*J*_OH ···F_ value for a conformationally restricted cyclohexane that predominantly assumes only one conformation (compound 2 in ref (38)). By doing so, we virtually eliminated the error that comes from the estimation of the populations. For this compound, we obtained a theoretical value (−16.5 Hz) that deviates considerably in magnitude from experiment (12.1 Hz),^116^ resulting in a relative error of 16.36%. As we did not find any correlation between the percentage of conformers with IMHBs and the error in the *J*-couplings for our set of γ-fluorohydrins, which if found could indicate an inability of the method to accurately calculate *J*-couplings for conformers with IMHBs, this result is surprising. Future work will focus on unraveling the source of this mismatch. Despite this, the excellent agreement obtained for the MP2/6-311++G(2d,p)/PCM data set leads us to believe that the protocol used to compute the *J*-coupling constants is sufficiently accurate to warrant a fair comparison between different data sets.

All in all, the NMR results here presented lead us to recommend that ANI-2x be used carefully in hybrid models for condensed-phase applications, especially for the modeling of compounds that have chemical interactions poorly described by ωB97X/6-31G* (e.g., hydrogen bonds). This conclusion is further supported by determining the *R*^2^ (0.86 vs 0.70) and RMSE (1.77 vs 3.45 Hz) values of GAFF.MOD-RESP/CHCl_3_ and ANI-2x-RESP/CHCl_3_, respectively, relative to the ωB97X/6-31G*/PCM NMR data. These results corroborate the findings regarding the sampling accuracy in chloroform solution (Figures 9 and 10), which indicate that GAFF.MOD-RESP/CHCl_3_ reproduces ωB97X/6-31G*/PCM better than ANI-2x-RESP/CHCl_3_.

## 4 Conclusions

We have presented a comparative study that evaluates the performance of an NNP (ANI-2x), a conventional FF (GAFF), and an optimally tuned FF (GAFF.MOD) relative to experimental and QM data. To this end, for a set of γ-fluorohydrins, we assessed the energetic and geometric agreement in the gas phase, the sampling accuracy in the gas phase and chloroform solution, and the accuracy of the estimates of the *J*-coupling constants relative to experimental data. The results and discussions presented highlight the strengths and weaknesses of each model, providing guidelines for use and future development of FFs and ML potentials. We believe that this study may have implications in different areas of chemistry and biology, especially for those interested in applications involving modeling of small organic compounds, which is very important for the drug design community. The main conclusions of this study are the following:

- nMC-MC simulations performed using ANI-2x as the approximate potential indicate a high similarity between this NNP and the level of theory it was trained to reproduce, ωB97X/6-31G*. These nMC-MC results also show that ANI-2x can produce stable MD simulations, with numerical stability comparable to that of GAFF-like FFs.
- In the gas phase, ANI-2x is the model that best reproduces the ωB97X/6-31G* energy landscape, followed by GAFF.MOD and GAFF. ANI-2x is also the model that best reproduces the energies and geometries of the gas-phase ωB97X/6-31G* minima, followed by GAFF.MOD and GAFF.
- The superior accuracy of ANI-2x in reproducing the ωB97X/6-31G* energy landscape does not always translate into higher sampling accuracy than GAFF.MOD, since ANI-2x shows similar performance to GAFF.MOD in this regard. Hence, while GAFF.MOD performs poorly in describing the minutiae of the ωB97X/6-31G* energy landscape, GAFF.MOD performs reasonably well in terms of relative energies, thus achieving similar performance to ANI-2x.
- ANI-2x excels in describing the minutiae of the ωB97X/6-31G* PES, especially in the gas phase. Nonetheless, ANI-2x also shows a tendency to predict stronger-than-expected hydrogen bonding and to overstabilize global minima, and it cannot properly capture dispersion interactions. These problems are related to energetic and entropic errors that mainly impact the relative populations between conformers, preventing ANI-2x from excelling in sampling accuracy.
- When MP2/6-311++G(2d,p) is the QM reference, GAFF stands out as the best-performing model in terms of both sampling accuracy and energetic agreement, followed by GAFF.MOD and ANI-2x.
- In chloroform solution, GAFF.MOD-RESP/CHCl_3_ is the model that predicts better sampling accuracy when ωB97X/6-31G*/PCM is used as the reference, significantly outperforming ANI-2x-RESP/CHCl_3_. The decrease in performance of the ANI-2x potential when used in a hybrid NNP/MM model suggests significant imbalances between the ligand–solvent intermolecular and ligand intramolecular interactions. Hence, the use of ANI-2x in an NNP/MM framework should be undertaken with caution due to the potential inconsistencies that may arise by directly combining available LJ 12–6 parameters with this NNP. Owing to their internal consistency, conventional and optimally tuned FFs remain the best models available for simulating condensed-phase systems.
- The NMR analysis revealed that MP2/6-311++G(2d,p)/PCM is the level of theory that best reproduces the experimental ^h1^*J*_OH···F_ coupling constants, followed by GAFF-RESP/CHCl_3_. These results suggest that MP2/6-311++G(2d,p) is closer to the experiment than ωB97X/6-31G*, and that GAFF is the model that best reproduces the experimental data. Furthermore, GAFF-RESP/CHCl_3_ is the model that best reproduces the MP2/6-311++G(2d,p)/PCM data, and GAFF.MOD-RESP/CHCl_3_ the model that best reproduces the ωB97X/6-31G*/PCM data. These observations corroborate the findings of the sampling accuracy in chloroform solution.

It is indisputable that ANI-2x has its merits, especially when it comes to modeling molecules in the gas phase. The merits of ANI-2x lie in a generally good description of the PES of small organic compounds. However, this study shows that some issues with using ANI-2x may have been overlooked in many applications. ANI-2x tends to predict stronger-than-expected hydrogen bonding and to overstabilize global minima, and it cannot properly capture dispersion interactions. These are observations that may limit the widespread use of ANI-2x in the long term, suggesting that an improved version of this NNP would be welcome. Furthermore, the use of ANI-2x in an NNP/MM framework should be undertaken with caution due to the potential inconsistencies that may arise. For some systems, directly combining ANI-2x with readily available FF parameters may lead to imbalances between different parts of the hybrid model, resulting in a significant decrease in performance. Because of their internal consistency, conventional and optimally tuned FFs remain the best models available for simulating condensed-phase systems. FFs also have the advantage of being computationally cheaper than NNPs. For NNP/MM models to become routinely used in condensed-phase simulations, sets of nonbonded MM parameters consistent with NNPs need to be derived; otherwise, the accuracy of NNP/MM models will always be compromised to some extent.

We must stress that all our of conclusions are based on the particular set of γ-fluorohydrins considered in this study. We cannot exclude that the observed performance may vary for other systems and that our conclusions therefore may not always be extrapolatable. In future work, it would be interesting to use the nMC-MC algorithm^21^ to generate QM/MM ensembles of structures against which we could compare the performance of the NNP/MM data. Despite the high computational cost that such calculations would entail, QM/MM structures could provide a better reference than the current QM/PCM level of theory used, which, for example, does not consider intermolecular interactions between the ligand and the (implicit) solvent. Moreover, to better understand the origin of the ANI-2x deficits observed, it would also be valuable to train an ANI-like model using QM energies and forces. In principle, this optimally tuned ANI-like model should outperform both ANI-2x and GAFF.MOD, thus proving the superiority of NNPs for modeling small organic molecules. Finally, it would also be interesting to optimize the nonbonded parameters for GAFF.MOD to attempt to achieve higher accuracy levels. It should be kept in mind, however, that if the goal is to use the optimized FFs in condensed-phase scenarios, care should be taken not to compromise the balance between intramolecular and intermolecular interactions.

## Data and Software Availability

The data used in this paper are available from the Zenodo repository at https://doi.org/10.5281/zenodo.7015273. ParaMol, the software used for the nMC-MC simulations and FF parametrization, can be found at https://github.com/JMorado/ParaMol. The OpenMM software and the openmm-ml plugin used to perform the simulations can be found at https://github.com/openmm/openmm and https://github.com/openmm/openmm-ml, respectively.

## Supporting Information Available

SMILES for the 10 γ-fluorohydrins (CSV)
Values of the *prior* widths and supporting figures (PDF)

## References

[ref1] Huggins D. J.; Biggin P. C.; Dämgen M. A.; Essex J. W.; Harris S. A.; Henchman R. H.; Khalid S.; Kuzmanic A.; Laughton C. A.; Michel J.; Mulholland A. J.; Rosta E.; Sansom M. S. P.; van der Kamp M. W. Biomolecular Simulations: From Dynamics and Mechanisms to Computational Assays of Biological Activity. Wiley Interdiscip. Rev.: Comput. Mol. Sci. 2019, 9, e1393 10.1002/wcms.1393.

[ref2] Hahn D. F.; Bayly C. I.; Boby M. L.; Bruce Macdonald H. E.; Chodera J. D.; Gapsys V.; Mey A. S. J. S.; Mobley D. L.; Benito L. P.; Schindler C. E. M.; Tresadern G.; Warren G. L. Best Practices for Constructing, Preparing, and Evaluating Protein-Ligand Binding Affinity Benchmarks [Article v1.0]. Living J. Comput. Mol. Sci. 2022, 4, 1497 10.33011/livecoms.4.1.1497. 36382113 PMC9662604

[ref3] Kashefolgheta S.; Oliveira M. P.; Rieder S. R.; Horta B. A. C.; Acree W. E.; Hünenberger P. H. Evaluating Classical Force Fields Against Experimental Cross-Solvation Free Energies. J. Chem. Theory Comput. 2020, 16, 7556–7580. 10.1021/acs.jctc.0c00688. 33147017

[ref4] Song L. F.; Merz K. M. Evolution of Alchemical Free Energy Methods in Drug Discovery. J. Chem. Inf. Model. 2020, 60, 5308–5318. 10.1021/acs.jcim.0c00547. 32818371

[ref5] Lee T.-S.; Allen B. K.; Giese T. J.; Guo Z.; Li P.; Lin C.; McGee T. D.; Pearlman D. A.; Radak B. K.; Tao Y.; Tsai H.-C.; Xu H.; Sherman W.; York D. M. Alchemical Binding Free Energy Calculations in AMBER20: Advances and Best Practices for Drug Discovery. J. Chem. Inf. Model. 2020, 60, 5595–5623. 10.1021/acs.jcim.0c00613. 32936637 PMC7686026

[ref6] Miller E. B.; Murphy R. B.; Sindhikara D.; Borrelli K. W.; Grisewood M. J.; Ranalli F.; Dixon S. L.; Jerome S.; Boyles N. A.; Day T.; Ghanakota P.; Mondal S.; Rafi S. B.; Troast D. M.; Abel R.; Friesner R. A. Reliable and Accurate Solution to the Induced Fit Docking Problem for Protein–Ligand Binding. J. Chem. Theory Comput. 2021, 17, 2630–2639. 10.1021/acs.jctc.1c00136. 33779166

[ref7] Huang S.-Y.; Zou X. Advances and Challenges in Protein-Ligand Docking. Int. J. Mol. Sci. 2010, 11, 3016–3034. 10.3390/ijms11083016. 21152288 PMC2996748

[ref8] Foloppe N.; Chen I.-J. Conformational Sampling and Energetics of Drug-Like Molecules. Curr. Med. Chem. 2009, 16, 3381–3413. 10.2174/092986709789057680. 19515013

[ref9] Foloppe N.; Chen I.-J. Towards Understanding the Unbound State of Drug Compounds: Implications for the Intramolecular Reorganization Energy Upon Bindinconformational Sampling and Energetics of Drug-Like Molecules. Bioorg. Med. Chem. 2016, 24, 2159–2189. 10.1016/j.bmc.2016.03.022. 27061672

[ref10] Hall R. J.; Mortenson P. N.; Murray C. W. Efficient Exploration of Chemical Space by Fragment-based Screening. Prog. Biophys. Mol. Biol. 2014, 116, 82–91. 10.1016/j.pbiomolbio.2014.09.007. 25268064

[ref11] Tamanini E.; Buck I. M.; Chessari G.; Chiarparin E.; Day J. E. H.; Frederickson M.; Griffiths-Jones C. M.; Hearn K.; Heightman T. D.; Iqbal A.; Johnson C. N.; Lewis E. J.; Martins V.; Peakman T.; Reader M.; Rich S. J.; Ward G. A.; Williams P. A.; Wilsher N. E. Discovery of a Potent Nonpeptidomimetic, Small-Molecule Antagonist of Cellular Inhibitor of Apoptosis Protein 1 (cIAP1) and X-Linked Inhibitor of Apoptosis Protein (XIAP). J. Med. Chem. 2017, 60, 4611–4625. 10.1021/acs.jmedchem.6b01877. 28492317

[ref12] Bruno A.; Costantino G.; Sartori L.; Radi M. The In Silico Drug Discovery Toolbox: Applications in Lead Discovery and Optimization. Curr. Med. Chem. 2019, 26, 3838–3873. 10.2174/0929867324666171107101035. 29110597

[ref13] Williams K.; Bilsland E.; Sparkes A.; Aubrey W.; Young M.; Soldatova L. N.; De Grave K.; Ramon J.; de Clare M.; Sirawaraporn W.; et al. Cheaper Faster Drug Development Validated by the Repositioning of Drugs Against Neglected Tropical Diseases. J. R. Soc., Interface 2015, 12, 20141289 10.1098/rsif.2014.1289. 25652463 PMC4345494

[ref14] Ferrero E.; Brachat S.; Jenkins J. L.; Marc P.; Skewes-Cox P.; Altshuler R. C.; Gubser Keller C.; Kauffmann A.; Sassaman E. K.; Laramie J. M.; et al. Ten Simple Rules to Power Drug Discovery with Data Science. PLoS Comput. Biol. 2020, 16, e1008126 10.1371/journal.pcbi.1008126. 32853229 PMC7451597

[ref15] Čížek J. On the Correlation Problem in Atomic and Molecular Systems. Calculation of Wavefunction Components in Ursell-Type Expansion Using Quantum-Field Theoretical Methods. J. Chem. Phys. 1966, 45, 4256–4266. 10.1063/1.1727484.

[ref16] Bartlett R. J.; Musiał M. Coupled-Cluster Theory in Quantum Chemistry. Rev. Mod. Phys. 2007, 79, 291–352. 10.1103/RevModPhys.79.291.

[ref17] Michel J.; Taylor R. D.; Essex J. W. Efficient Generalized Born Models for Monte Carlo Simulations. J. Chem. Theory Comput. 2006, 2, 732–739. 10.1021/ct600069r. 26626678

[ref18] Sampson C.; Fox T.; Tautermann C. S.; Woods C.; Skylaris C.-K. A “Stepping Stone” Approach for Obtaining Quantum Free Energies of Hydration. J. Phys. Chem. B 2015, 119, 7030–7040. 10.1021/acs.jpcb.5b01625. 25985723

[ref19] Rosa M.; Micciarelli M.; Laio A.; Baroni S. Sampling Molecular Conformers in Solution with Quantum Mechanical Accuracy at a Nearly Molecular-Mechanics Cost. J. Chem. Theory Comput. 2016, 12, 4385–4389. 10.1021/acs.jctc.6b00470. 27494227

[ref20] Cave-Ayland C.; Skylaris C.-K.; Essex J. W. A Monte Carlo Resampling Approach for the Calculation of Hybrid Classical and Quantum Free Energies. J. Chem. Theory Comput. 2017, 13, 415–424. 10.1021/acs.jctc.6b00506. 28029794

[ref21] Morado J.; Mortenson P. N.; Nissink J. W. M.; Verdonk M. L.; Ward R. A.; Essex J. W.; Skylaris C.-K. Generation of Quantum Configurational Ensembles Using Approximate Potentials. J. Chem. Theory Comput. 2021, 17, 7021–7042. 10.1021/acs.jctc.1c00532. 34644088

[ref22] Vanommeslaeghe K.; MacKerell A. CHARMM Additive and Polarizable Force Fields for Biophysics and Computer-Aided Drug Design. Biochim. Biophys. Acta, Gen. Subj. 2015, 1850, 861–871. 10.1016/j.bbagen.2014.08.004. PMC4334745 25149274

[ref23] Pierce L. C. T.; Salomon-Ferrer R.; de Oliveira C. A. F.; McCammon J. A.; Walker R. C. Routine Access to Millisecond Time Scale Events with Accelerated Molecular Dynamics. J. Chem. Theory Comput 2012, 8, 2997–3002. 10.1021/ct300284c. 22984356 PMC3438784

[ref24] Lim V. T.; Hahn D. F.; Tresadern G.; Bayly C. I.; Mobley D. L. Benchmark Assessment of Molecular Geometries and Energies from Small Molecule Force Fields. F1000Research 2020, 9, 1390 10.12688/f1000research.27141.1. PMC7863993 33604023

[ref25] Ehrman J. N.; Lim V. T.; Bannan C. C.; Thi N.; Kyu D. Y.; Mobley D. L. Improving Small Molecule Force Fields by Identifying and Characterizing Small Molecules with Inconsistent Parameters. J. Comput.-Aided Mol. Des. 2021, 35, 271–284. 10.1007/s10822-020-00367-1. 33506360 PMC8162916

[ref26] Morado J.; Mortenson P. N.; Verdonk M. L.; Ward R. A.; Essex J. W.; Skylaris C.-K. ParaMol: A Package for Automatic Parameterization of Molecular Mechanics Force Fields. J. Chem. Inf. Model. 2021, 61, 2026–2047. 10.1021/acs.jcim.0c01444. 33750120

[ref27] Qiu Y.; Smith D. G. A.; Boothroyd S.; Jang H.; Hahn D. F.; Wagner J.; Bannan C. C.; Gokey T.; Lim V. T.; Stern C. D.; Rizzi A.; Tjanaka B.; Tresadern G.; Lucas X.; Shirts M. R.; Gilson M. K.; Chodera J. D.; Bayly C. I.; Mobley D. L.; Wang L.-P. Development and Benchmarking of Open Force Field v1.0.0—the Parsley Small-Molecule Force Field. J. Chem. Theory Comput. 2021, 17, 6262–6280. 10.1021/acs.jctc.1c00571. 34551262 PMC8511297

[ref28] Kocer E.; Ko T. W.; Behler J. Neural Network Potentials: A Concise Overview of Methods. Annu. Rev. Phys. Chem. 2022, 73, 163–186. 10.1146/annurev-physchem-082720-034254. 34982580

[ref29] Unke O. T.; Chmiela S.; Sauceda H. E.; Gastegger M.; Poltavsky I.; Schütt K. T.; Tkatchenko A.; Müller K.-R. Machine Learning Force Fields. Chem. Rev. 2021, 121, 10142–10186. 10.1021/acs.chemrev.0c01111. 33705118 PMC8391964

[ref30] Pinheiro M.; Ge F.; Ferré N.; Dral P. O.; Barbatti M. Choosing the Right Molecular Machine Learning Potential. Chem. Sci. 2021, 12, 14396–14413. 10.1039/D1SC03564A. 34880991 PMC8580106

[ref31] Zubatiuk T.; Isayev O. Development of Multimodal Machine Learning Potentials: Toward a Physics-Aware Artificial Intelligence. Acc. Chem. Res. 2021, 54, 1575–1585. 10.1021/acs.accounts.0c00868. 33715355

[ref32] Yoo P.; Sakano M.; Desai S.; Islam M. M.; Liao P.; Strachan A. Neural Network Reactive Force Field for C, H, N, and O Systems. npj Comput. Mater. 2021, 7, 9 10.1038/s41524-020-00484-3.

[ref33] Christensen A. S.; Bratholm L. A.; Faber F. A.; Anatole Von Lilienfeld O. FCHL Revisited: Faster and More Accurate Quantum Machine Learning. J. Chem. Phys. 2020, 152, 044107 10.1063/1.5126701. 32007071

[ref34] Chmiela S.; Tkatchenko A.; Sauceda H. E.; Poltavsky I.; Schütt K. T.; Müller K.-R. Machine Learning of Accurate Energy-Conserving Molecular Force Fields. Sci. Adv. 2017, 3, e1603015 10.1126/sciadv.1603015. 28508076 PMC5419702

[ref35] Smith J. S.; Isayev O.; Roitberg A. E. ANI-1: An Extensible Neural Network Potential with DFT Accuracy at Force Field Computational Cost. Chem. Sci. 2017, 8, 3192–3203. 10.1039/C6SC05720A. 28507695 PMC5414547

[ref36] Schütt K. T.; Sauceda H. E.; Kindermans P.-J.; Tkatchenko A.; Müller K.-R. SchNet – A Deep Learning Architecture for Molecules and Materials. J. Chem. Phys. 2018, 148, 241722 10.1063/1.5019779. 29960322

[ref37] Unke O. T.; Meuwly M. PhysNet: A Neural Network for Predicting Energies, Forces, Dipole Moments, and Partial Charges. J. Chem. Theory Comput. 2019, 15, 3678–3693. 10.1021/acs.jctc.9b00181. 31042390

[ref38] Linclau B.; Peron F.; Bogdan E.; Wells N.; Wang Z.; Compain G.; Fontenelle C. Q.; Galland N.; Le Questel J.-Y.; Graton J. Intramolecular OH-Fluorine Hydrogen Bonding in Saturated, Acyclic Fluorohydrins: The γ-Fluoropropanol Motif. Chem. - Eur. J. 2015, 21, 17808–17816. 10.1002/chem.201503253. 26494542 PMC4676915

[ref39] Devereux C.; Smith J. S.; Huddleston K. K.; Barros K.; Zubatyuk R.; Isayev O.; Roitberg A. E. Extending the Applicability of the ANI Deep Learning Molecular Potential to Sulfur and Halogens. J. Chem. Theory Comput. 2020, 16, 4192–4202. 10.1021/acs.jctc.0c00121. 32543858

[ref40] Smith J. S.; Nebgen B.; Lubbers N.; Isayev O.; Roitberg A. E. Less Is More: Sampling Chemical Space with Active Learning. J. Chem. Phys. 2018, 148, 241733 10.1063/1.5023802. 29960353

[ref41] Smith J. S.; Nebgen B. T.; Zubatyuk R.; Lubbers N.; Devereux C.; Barros K.; Tretiak S.; Isayev O.; Roitberg A. E. Approaching Coupled Cluster Accuracy with a General-Purpose Neural Network Potential Through Transfer Learning. Nat. Commun. 2019, 10, 2903 10.1038/s41467-019-10827-4. 31263102 PMC6602931

[ref42] Smith J. S.; Isayev O.; Roitberg A. E. ANI-1, A Data Set of 20 Million Calculated off-Equilibrium Conformations for Organic Molecules. Sci. Data 2017, 4, 170193 10.1038/sdata.2017.193. 29257127 PMC5735918

[ref43] Smith J. S.; Zubatyuk R.; Nebgen B.; Lubbers N.; Barros K.; Roitberg A. E.; Isayev O.; Tretiak S. The ANI-1ccx and ANI-1x Data Sets, Coupled-Cluster and Density Functional Theory Properties for Molecules. Sci. Data 2020, 7, 134 10.1038/s41597-020-0473-z. 32358545 PMC7195467

[ref44] Chai J.-D.; Head-Gordon M. Systematic Optimization of Long-Range Corrected Hybrid Density Functionals. J. Chem. Phys. 2008, 128, 084106 10.1063/1.2834918. 18315032

[ref45] Zheng P.; Zubatyuk R.; Wu W.; Isayev O.; Dral P. O. Artificial Intelligence-Enhanced Quantum Chemical Method with Broad Applicability. Nat. Commun. 2021, 12, 7022 10.1038/s41467-021-27340-2. 34857738 PMC8640006

[ref46] Zeng J.; Giese T. J.; Ekesan Ş.; York D. M. Development of Range-Corrected Deep Learning Potentials for Fast, Accurate Quantum Mechanical/Molecular Mechanical Simulations of Chemical Reactions in Solution. J. Chem. Theory Comput. 2021, 17, 6993–7009. 10.1021/acs.jctc.1c00201. 34644071 PMC8578402

[ref47] Giese T. J.; Zeng J.; Ekesan Ş.; York D. M. Combined QM/MM, Machine Learning Path Integral Approach to Compute Free Energy Profiles and Kinetic Isotope Effects in RNA Cleavage Reactions. J. Chem. Theory Comput. 2022, 18, 4304–4317. 10.1021/acs.jctc.2c00151. 35709391 PMC9283286

[ref48] Pan X.; Yang J.; Van R.; Epifanovsky E.; Ho J.; Huang J.; Pu J.; Mei Y.; Nam K.; Shao Y. Machine-Learning-Assisted Free Energy Simulation of Solution-Phase and Enzyme Reactions. J. Chem. Theory Comput. 2021, 17, 5745–5758. 10.1021/acs.jctc.1c00565. 34468138 PMC9070000

[ref49] Lahey S.-L. J.; Thien Phuc T. N.; Rowley C. N. Benchmarking Force Field and the ANI Neural Network Potentials for the Torsional Potential Energy Surface of Biaryl Drug Fragments. J. Chem. Inf. Model. 2020, 60, 6258–6268. 10.1021/acs.jcim.0c00904. 33263401

[ref50] Folmsbee D. L.; Koes D. R.; Hutchison G. R. Evaluation of Thermochemical Machine Learning for Potential Energy Curves and Geometry Optimization. J. Phys. Chem. A 2021, 125, 1987–1993. 10.1021/acs.jpca.0c10147. 33630611

[ref51] Rosenberger D.; Smith J. S.; Garcia A. E. Modeling of Peptides with Classical and Novel Machine Learning Force Fields: A Comparison. J. Phys. Chem. B 2021, 125, 3598–3612. 10.1021/acs.jpcb.0c10401. 33798336

[ref52] Temel M.; Tayfuroglu O.; Kocak A. The Performance of ANI-ML potentials for Ligand-n(H_2_O) Interaction Energies and Estimation of Hydration Free energies From eEnd-point MD Simulations. J. Comput. Chem. 2023, 44, 559–569. 10.1002/jcc.27022. 36324248

[ref53] Jiang S.; Liu Y.; Wang C.; Huang T. Benchmarking General Neural Network Potential ANI-2x on Aerosol Nucleation Molecular Clusters. Int. J. Quantum Chem. 2023, 10.1002/qua.27087.

[ref54] Galvelis R.; Doerr S.; Damas J. M.; Harvey M. J.; De Fabritiis G. A Scalable Molecular Force Field Parameterization Method Based on Density Functional Theory and Quantum-Level Machine Learning. J. Chem. Inf. Model. 2019, 59, 3485–3493. 10.1021/acs.jcim.9b00439. 31322877

[ref55] Wang J.; Wolf R. M.; Caldwell J. W.; Kollman P. A.; Case D. A. Development and Testing of a General Amber Force Field. J. Comput. Chem. 2004, 25, 1157–1174. 10.1002/jcc.20035. 15116359

[ref56] Zhu S. Validation of the Generalized Force Fields GAFF, CGenFF, OPLS-AA, and PRODRGFF by Testing Against Experimental Osmotic Coefficient Data for Small Drug-Like Molecules. J. Chem. Inf. Model. 2019, 59, 4239–4247. 10.1021/acs.jcim.9b00552. 31557024

[ref57] Vreven T.; Morokuma K. Hybrid Methods: ONIOM(QM:MM) and QM/MM. Annu. Rep. Comput. Chem. 2006, 2, 35–51. 10.1016/S1574-1400(06)02003-2.

[ref58] Lahey S.-L. J.; Rowley C. N. Simulating Protein–Ligand Binding with Neural Network Potentials. Chem. Sci. 2020, 11, 2362–2368. 10.1039/C9SC06017K. 34084397 PMC8157423

[ref59] Vant J. W.; Lahey S.-L. J.; Jana K.; Shekhar M.; Sarkar D.; Munk B. H.; Kleinekathöfer U.; Mittal S.; Rowley C.; Singharoy A. Flexible Fitting of Small Molecules into Electron Microscopy Maps Using Molecular Dynamics Simulations with Neural Network Potentials. J. Chem. Inf. Model. 2020, 60, 2591–2604. 10.1021/acs.jcim.9b01167. 32207947 PMC7311632

[ref60] Cole D. J.; Mones L.; Csányi G. A machine learning based intramolecular potential for a flexible organic molecule. Faraday Discuss. 2020, 224, 247–264. 10.1039/D0FD00028K. 32955056

[ref61] Wieder M.; Fass J.; Chodera J. D. Fitting Quantum Machine Learning Potentials to Experimental Free Energy Data: Predicting Tautomer Ratios in Solution. Chem. Sci. 2021, 12, 11364–11381. 10.1039/D1SC01185E. 34567495 PMC8409483

[ref62] Fink T.; Reymond J.-L. Virtual Exploration of the Chemical Universe up to 11 Atoms of C, N, O, F: Assembly of 26.4 Million Structures (110.9 Million Stereoisomers) and Analysis for New Ring Systems, Stereochemistry, Physicochemical Properties, Compound Classes, and Drug Discovery. J. Chem. Inf. Model. 2007, 47, 342–353. 10.1021/ci600423u. 17260980

[ref63] Fink T.; Bruggesser H.; Reymond J.-L. Virtual Exploration of the Small-Molecule Chemical Universe below 160 Da. Angew. Chem., Int. Ed. 2005, 44, 1504–1508. 10.1002/anie.200462457. 15674983

[ref64] Bento A. P.; Gaulton A.; Hersey A.; Bellis L. J.; Chambers J.; Davies M.; Krüger F. A.; Light Y.; Mak L.; McGlinchey S.; Nowotka M.; Papadatos G.; Santos R.; Overington J. P. The ChEMBL Bioactivity Database: an Update. Nucleic Acids Res. 2014, 42, D1083–D1090. 10.1093/nar/gkt1031. 24214965 PMC3965067

[ref65] Brauer B.; Kesharwani M. K.; Kozuch S.; Martin J. M. L. The s66 × 8 Benchmark for Noncovalent Interactions Revisited: Explicitly Correlated Ab Initio Methods and Density Functional Theory. Phys. Chem. Chem. Phys. 2016, 18, 20905–20925. 10.1039/C6CP00688D. 26950084

[ref66] Landrum G. RDKit: Open-source cheminformatics. http://www.rdkit.org (accessed 2022-08-01).

[ref67] Elton D. C.; Boukouvalas Z.; Butrico M. S.; Fuge M. D.; Chung P. W. Applying Machine Learning Techniques to Predict the Properties of Energetic Materials. Sci. Rep. 2018, 8, 9059 10.1038/s41598-018-27344-x. 29899464 PMC5998124

[ref68] Pronobis W.; Tkatchenko A.; Müller K.-R. Many-Body Descriptors for Predicting Molecular Properties with Machine Learning: Analysis of Pairwise and Three-Body Interactions in Molecules. J. Chem. Theory Comput. 2018, 14, 2991–3003. 10.1021/acs.jctc.8b00110. 29750522

[ref69] Pinheiro M.; Ge F.; Ferré N.; Dral P. O.; Barbatti M. Choosing the Right Molecular Machine Learning Potential. Chem. Sci. 2021, 12, 14396–14413. 10.1039/D1SC03564A. 34880991 PMC8580106

[ref70] Rupp M.; Tkatchenko A.; Müller K.-R.; von Lilienfeld O. A. Fast and Accurate Modeling of Molecular Atomization Energies with Machine Learning. Phys. Rev. Lett. 2012, 108, 058301 10.1103/PhysRevLett.108.058301. 22400967

[ref71] Montavon G.; Hansen K.; Fazli S.; Rupp M.; Biegler F.; Ziehe A.; Tkatchenko A.; Lilienfeld A.; Müller K.-R. Learning Invariant Representations of Molecules for Atomization Energy Prediction. In Advances in Neural Information Processing Systems 25 (NIPS 2012); Curran Associates, 2012.

[ref72] Faber F.; Lindmaa A.; von Lilienfeld O. A.; Armiento R. Crystal Structure Representations for Machine Learning Models of Formation Energies. Int. J. Quantum Chem. 2015, 115, 1094–1101. 10.1002/qua.24917.

[ref73] Hansen K.; Biegler F.; Ramakrishnan R.; Pronobis W.; von Lilienfeld O. A.; Müller K.-R.; Tkatchenko A. Machine Learning Predictions of Molecular Properties: Accurate Many-Body Potentials and Nonlocality in Chemical Space. J. Phys. Chem. Lett. 2015, 6, 2326–2331. 10.1021/acs.jpclett.5b00831. 26113956 PMC4476293

[ref74] Bartók A. P.; Payne M. C.; Kondor R.; Csányi G. Gaussian Approximation Potentials: The Accuracy of Quantum Mechanics, without the Electrons. Phys. Rev. Lett. 2010, 104, 136403 10.1103/PhysRevLett.104.136403. 20481899

[ref75] Bartók A. P.; Kondor R.; Csányi G. On Representing Chemical Environments. Phys. Rev. B 2013, 87, 184115 10.1103/PhysRevB.87.184115.

[ref76] Bartók A. P.; De S.; Poelking C.; Bernstein N.; Kermode J. R.; Csányi G.; Ceriotti M. Machine Learning Unifies the Modeling of Materials and Molecules. Sci. Adv. 2017, 3, e1701816 10.1126/sciadv.1701816. 29242828 PMC5729016

[ref77] Behler J.; Parrinello M. Generalized Neural-Network Representation of High-Dimensional Potential-Energy Surfaces. Phys. Rev. Lett. 2007, 98, 146401 10.1103/PhysRevLett.98.146401. 17501293

[ref78] Besler B. H.; Merz K. M.; Kollman P. A. Atomic Charges Derived From Semiempirical Methods. J. Comput. Chem. 1990, 11, 431–439. 10.1002/jcc.540110404.

[ref79] Reynolds C. A.; Essex J. W.; Richards W. G. Atomic Charges for Variable Molecular Conformations. J. Am. Chem. Soc. 1992, 114, 9075–9079. 10.1021/ja00049a045.

[ref80] Bayly C. I.; Cieplak P.; Cornell W.; Kollman P. A. A Well-Behaved Electrostatic Potential Based Method Using Charge Restraints for Deriving Atomic Charges: The RESP Model. J. Phys. Chem. 1993, 97, 10269–10280. 10.1021/j100142a004.

[ref81] Cornell W. D.; Cieplak P.; Bayly C. I.; Kollman P. A. Application of RESP Charges to Calculate Conformational Energies, Hydrogen Bond Energies, and Free Energies of Solvation. J. Am. Chem. Soc. 1993, 115, 9620–9631. 10.1021/ja00074a030.

[ref82] Woods R.; Chappelle R. Restrained Electrostatic Potential Atomic Partial Charges for Condensed-Phase Simulations of Carbohydrates. J. Mol. Struct.: THEOCHEM 2000, 527, 149–156. 10.1016/S0166-1280(00)00487-5. PMC4191892 25309012

[ref83] Iftimie R.; Salahub D.; Wei D.; Schofield J. Using a Classical Potential as an Efficient Importance Function for Sampling from an Ab Initio Potential. J. Chem. Phys. 2000, 113, 4852 10.1063/1.1289534.

[ref84] Gelb L. D. Monte Carlo Simulations Using Sampling from an Approximate Potential. J. Chem. Phys. 2003, 118, 7747–7750. 10.1063/1.1563597.

[ref85] Turney J. M.; Simmonett A. C.; Parrish R. M.; Hohenstein E. G.; Evangelista F. A.; Fermann J. T.; Mintz B. J.; Burns L. A.; Wilke J. J.; Abrams M. L.; Russ N. J.; Leininger M. L.; Janssen C. L.; Seidl E. T.; Allen W. D.; Schaefer H. F.; King R. A.; Valeev E. F.; Sherrill C. D.; Crawford T. D. Psi4: An Open-Source Ab Initio Electronic Structure Program. Wiley Interdiscip. Rev.: Comput. Mol. Sci. 2012, 2, 556–565. 10.1002/wcms.93.

[ref86] Duane S.; Kennedy A.; Pendleton B. J.; Roweth D. Hybrid Monte Carlo. Phys. Lett. B 1987, 195, 216–222. 10.1016/0370-2693(87)91197-X.

[ref87] Guvench O.; MacKerell A. D. Automated conformational energy fitting for force-field development. J. Mol. Model. 2008, 14, 667–679. 10.1007/s00894-008-0305-0. 18458967 PMC2864003

[ref88] Hopkins C. W.; Roitberg A. E. Fitting of Dihedral Terms in Classical Force Fields as an Analytic Linear Least-Squares Problem. J. Chem. Inf. Model. 2014, 54, 1978–1986. 10.1021/ci500112w. 24960267

[ref89] Wang L.-P.; Van Voorhis T. Communication: Hybrid Ensembles for Improved Force Matching. J. Chem. Phys. 2010, 133, 231101 10.1063/1.3519043. 21186847

[ref90] Cieplak P.; Caldwell J.; Kollman P. Molecular Mechanical Models for Organic and Biological Systems Going Beyond the Atom Centered Two Body Additive Approximation: Aqueous Solution Free Energies of Methanol and *N*-Methyl Acetamide, Nucleic Acid Base, and Amide Hydrogen Bonding and Chloroform/Water Partition Coefficients of the Nucleic Acid Bases. J. Comput. Chem. 2001, 22, 1048–1057. 10.1002/jcc.1065.

[ref91] Åqvist J.; Wennerström P.; Nervall M.; Bjelic S.; Brandsdal B. O. Molecular Dynamics Simulations of Water and Biomolecules with a Monte Carlo Constant Pressure Algorithm. Chem. Phys. Lett. 2004, 384, 288–294. 10.1016/j.cplett.2003.12.039.

[ref92] Chow K.-H.; Ferguson D. M. Isothermal-Isobaric Molecular Dynamics Simulations with Monte Carlo Volume Sampling. Comput. Phys. Commun. 1995, 91, 283–289. 10.1016/0010-4655(95)00059-O.

[ref93] Darden T.; York D.; Pedersen L. Particle Mesh Ewald: An *N* log(*N*) Method for Ewald Sums in Large Systems. J. Chem. Phys. 1993, 98, 10089–10092. 10.1063/1.464397.

[ref94] Essmann U.; Perera L.; Berkowitz M. L.; Darden T.; Lee H.; Pedersen L. G. A Smooth Particle Mesh Ewald Method. J. Chem. Phys. 1995, 103, 8577–8593. 10.1063/1.470117.

[ref95] Eastman P.; Swails J.; Chodera J. D.; McGibbon R. T.; Zhao Y.; Beauchamp K. A.; Wang L.-P.; Simmonett A. C.; Harrigan M. P.; Stern C. D.; et al. OpenMM 7: Rapid Development of High Performance Algorithms for Molecular Dynamics. PLoS Comput. Biol. 2017, 13, e1005659 10.1371/journal.pcbi.1005659. 28746339 PMC5549999

[ref96] Wang J.; Wang W.; Kollman P. A.; Case D. A. Automatic Atom Type and Bond Type Perception in Molecular Mechanical Calculations. J. Mol. Graphics Modell. 2006, 25, 247–260. 10.1016/j.jmgm.2005.12.005. 16458552

[ref97] Flory P. J. Statistical Mechanics of Chain Molecules; Interscience Publishers, 1969.

[ref98] Benedetti E.; Morelli G.; Némethy G.; Scheraga H. A. Statistical and Energetic Analysis of Side-Chain Conformations in Oligopeptides. Int. J. Pept. Protein Res. 1983, 22, 1–15. 10.1111/j.1399-3011.1983.tb02062.x. 6885244

[ref99] Frisch M. J.; Trucks G. W.; Schlegel H. B.; Scuseria G. E.; Robb M. A.; Cheeseman J. R.; Scalmani G.; Barone V.; Mennucci B.; Petersson G. A.; Nakatsuji H.; Caricato M.; Li X.; Hratchian H. P.; Izmaylov A. F.; Bloino J.; Zheng G.; Sonnenberg J. L.; Hada M.; Ehara M.; Toyota K.; Fukuda R.; Hasegawa J.; Ishida M.; Nakajima T.; Honda Y.; Kitao O.; Nakai H.; Vreven T.; Montgomery J. A. Jr.; Peralta J. E.; Ogliaro F.; Bearpark M.; Heyd J. J.; Brothers E.; Kudin K. N.; Staroverov V. N.; Kobayashi R.; Normand J.; Raghavachari K.; Rendell A.; Burant J. C.; Iyengar S. S.; Tomasi J.; Cossi M.; Rega N.; Millam J. M.; Klene M.; Knox J. E.; Cross J. B.; Bakken V.; Adamo C.; Jaramillo J.; Gomperts R.; Stratmann R. E.; Yazyev O.; Austin A. J.; Cammi R.; Pomelli C.; Ochterski J. W.; Martin R. L.; Morokuma K.; Zakrzewski V. G.; Voth G. A.; Salvador P.; Dannenberg J. J.; Dapprich S.; Daniels A. D.; Farkas Ö.; Foresman J. B.; Ortiz J. V.; Cioslowski J.; Fox D. J. Gaussian 09, rev. D.01; Gaussian, Inc.: Wallingford, CT, 2009.

[ref100] Hjorth Larsen A.; Mortensen J. J.; Blomqvist J.; Castelli I. E.; Christensen R.; Dułak M.; Friis J.; Groves M. N.; Hammer B.; Hargus C.; Hermes E. D.; Jennings P. C.; Jensen P. B.; Kermode J.; Kitchin J. R.; Kolsbjerg E. L.; Kubal J.; Kaasbjerg K.; Lysgaard S.; Maronsson J. B.; Maxson T.; Olsen T.; Pastewka L.; Peterson A.; Rostgaard C.; Schiøtz J.; Schütt O.; Strange M.; Thygesen K. S.; Vegge T.; Vilhelmsen L.; Walter M.; Zeng Z.; Jacobsen K. W. The Atomic Simulation Environment - a Python Library for Working with Atoms. J. Phys.: Condens. Matter 2017, 29, 273002 10.1088/1361-648X/aa680e. 28323250

[ref101] Ditchfield R. Self-Consistent Perturbation Theory of Diamagnetism: I. A Gauge-Invariant LCAO Method for N.M.R. Chemical Shifts. Mol. Phys. 1974, 27, 789–807. 10.1080/00268977400100711.

[ref102] Wolinski K.; Hinton J. F.; Pulay P. Efficient Implementation of the Gauge-independent Atomic Orbital Method for NMR Chemical Shift Calculations. J. Am. Chem. Soc. 1990, 112, 8251–8260. 10.1021/ja00179a005.

[ref103] Helgaker T.; Watson M.; Handy N. C. Analytical Calculation of Nuclear Magnetic Resonance Indirect Spin–spin Coupling Constants at the Generalized Gradient Approximation and Hybrid Levels of Density-functional Theory. J. Chem. Phys. 2000, 113, 9402–9409. 10.1063/1.1321296.

[ref104] Wilson P. J.; Bradley T. J.; Tozer D. J. Hybrid Exchange-Correlation Functional Determined From Thermochemical Data And Ab Initio Potentials. J. Chem. Phys. 2001, 115, 9233–9242. 10.1063/1.1412605.

[ref105] Jensen F. The Basis Set Convergence of Spin-Spin Coupling Constants Calculated by Density Functional Methods. J. Chem. Theory Comput. 2006, 2, 1360–1369. 10.1021/ct600166u. 26626843

[ref106] Keal T. W.; Helgaker T.; Sałek P.; Tozer D. J. Choice of Exchange-Correlation Functional for Computing NMR Indirect Spin–Spin Coupling Constants. Chem. Phys. Lett. 2006, 425, 163–166. 10.1016/j.cplett.2006.05.032.

[ref107] Kupka T.; Nieradka M.; Stachów M.; Pluta T.; Nowak P.; Kjær H.; Kongsted J.; Kaminsky J. Basis Set Convergence of Indirect Spin–Spin Coupling Constants in the Kohn–Sham Limit for Several Small Molecules. J. Phys. Chem. A 2012, 116, 3728–3738. 10.1021/jp212588h. 22401301

[ref108] Kristyán S.; Pulay P. Can (Semi)Local Density Functional Theory Account For the London Dispersion Forces?. Chem. Phys. Lett. 1994, 229, 175–180. 10.1016/0009-2614(94)01027-7.

[ref109] Dobson J. F.; McLennan K.; Rubio A.; Wang J.; Gould T.; Le H. M.; Dinte B. P. Prediction of Dispersion Forces: Is There a Problem?. Aust. J. Chem. 2001, 54, 513 10.1071/CH01052.

[ref110] Chai J.-D.; Head-Gordon M. Long-Range Corrected Hybrid Density Functionals with Damped Atom–Atom Dispersion Corrections. Phys. Chem. Chem. Phys. 2008, 10, 6615 10.1039/b810189b. 18989472

[ref111] Lin Y.-S.; Li G.-D.; Mao S.-P.; Chai J.-D. Long-Range Corrected Hybrid Density Functionals with Improved Dispersion Corrections. J. Chem. Theory Comput. 2013, 9, 263–272. 10.1021/ct300715s. 26589028

[ref112] Ochterski J. W. Thermochemistry in Gaussian; Gaussian, Inc., 2000; https://gaussian.com/thermo/ (accessed 2023-02-18).

[ref113] Leach A. R. A Survey of Methods for Searching the Conformational Space of Small and Medium-Sized Molecules. Rev. Comput. Chem. 2007, 2, 1–55. 10.1002/9780470125793.ch1.

[ref114] Pracht P.; Grimme S. Calculation of Absolute Molecular Entropies and Heat Capacities Made Simple. Chem. Sci. 2021, 12, 6551–6568. 10.1039/D1SC00621E. 34040731 PMC8139639

[ref115] Grimme S.; Antony J.; Ehrlich S.; Krieg H. A Consistent and Accurate Ab Initio Parametrization of Density Functional Dispersion Correction (DFT-D) for the 94 Elements H-Pu. J. Chem. Phys. 2010, 132, 154104 10.1063/1.3382344. 20423165

[ref116] Graton J.; Wang Z.; Brossard A.-M.; Gonçalves Monteiro D.; Le Questel J.-Y.; Linclau B. An Unexpected and Significantly Lower Hydrogen-Bond-Donating Capacity of Fluorohydrins Compared to Nonfluorinated Alcohols. Angew. Chem., Int. Ed. 2012, 51, 6176–6180. 10.1002/anie.201202059. PMC3601419 22577052

