# Supplementary material for: Does a Machine-Learned Potential Perform Better Than an Optimally Tuned Traditional Force Field? A Case Study on Fluorohydrins
Source: J Chem Inf Model. 2023 Apr 18;63(9):2810–27. doi: 10.1021/acs.jcim.2c01510 (PMC10170518; doi:10.1021/acs.jcim.2c01510)
Supplement: Supplementary file 2 — ci2c01510_si_002.pdf [file ci2c01510_si_002.pdf]

Supporting Information

Does a Machine-Learnt Potential Perform Better Than an Optimally Tuned Traditional Force Field? A Case Study on Fluorohydrins

João Morado,† Paul N. Mortenson,‡ J. Willem M. Nissink,¶ Jonathan W. Essex,*,† and Chris-Kriton Skylaris*,†

†*School of Chemistry, University of Southampton, Highfield, Southampton SO17 1BJ, United Kingdom*

‡*Astex Pharmaceuticals, 436 Cambridge Science Park, Milton Road, Cambridge CB4 0QA, United Kingdom*

¶*Computational Chemistry, Oncology R&D, AstraZeneca, Cambridge CB4 0WG, UK*

E-mail: j.w.essex@soton.ac.uk; c.skylaris@soton.ac.uk

Prior Widths

Table S1: ParaMol default prior width values for each parameter type.

**Parameter type**	**Prior width**
bond length	0.05 nm
bond force constant	10^5 kJ mol^{-1} nm^{-2}
bond angle	$\pi/16$ rad
angle force constant	10^2 kJ mol^{-1} rad^{-2}
dihedral phase	π rad
dihedral amplitude	16.736 kJ mol^{-1}
Lennard-Jones 12-6 ϵ	0.30 kJ mol^{-1}
Lennard-Jones 12-6 σ	0.20 nm
charge	0.5 e
1–4 electrostatic scaling factor	1.0
1–4 Lennard-Jones scaling factor	1.0

Supporting Figures

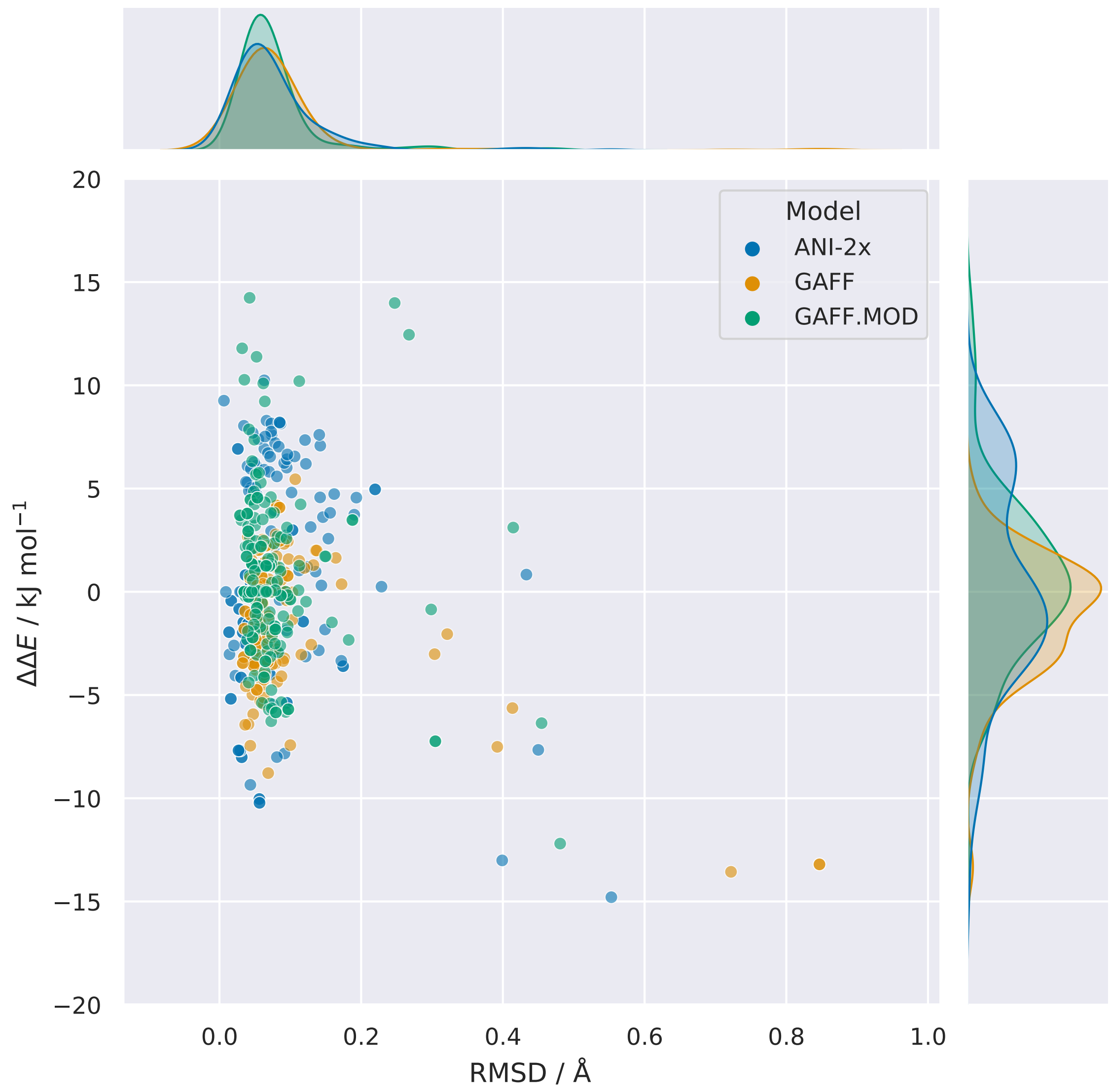

Figure S1: Scatter plots of the relative conformer energies ($\Delta\Delta E$) versus the RMSD of atomic positions. Each point was obtained by performing a geometry optimization using GAFF, GAFF.MOD, or ANI-2x, starting from all QM minima within 12.552 kJ mol^{-1} (3 kcal mol^{-1}) from the global minimum. The QM reference is the MP2/6-311++G(2d,p) level of theory.

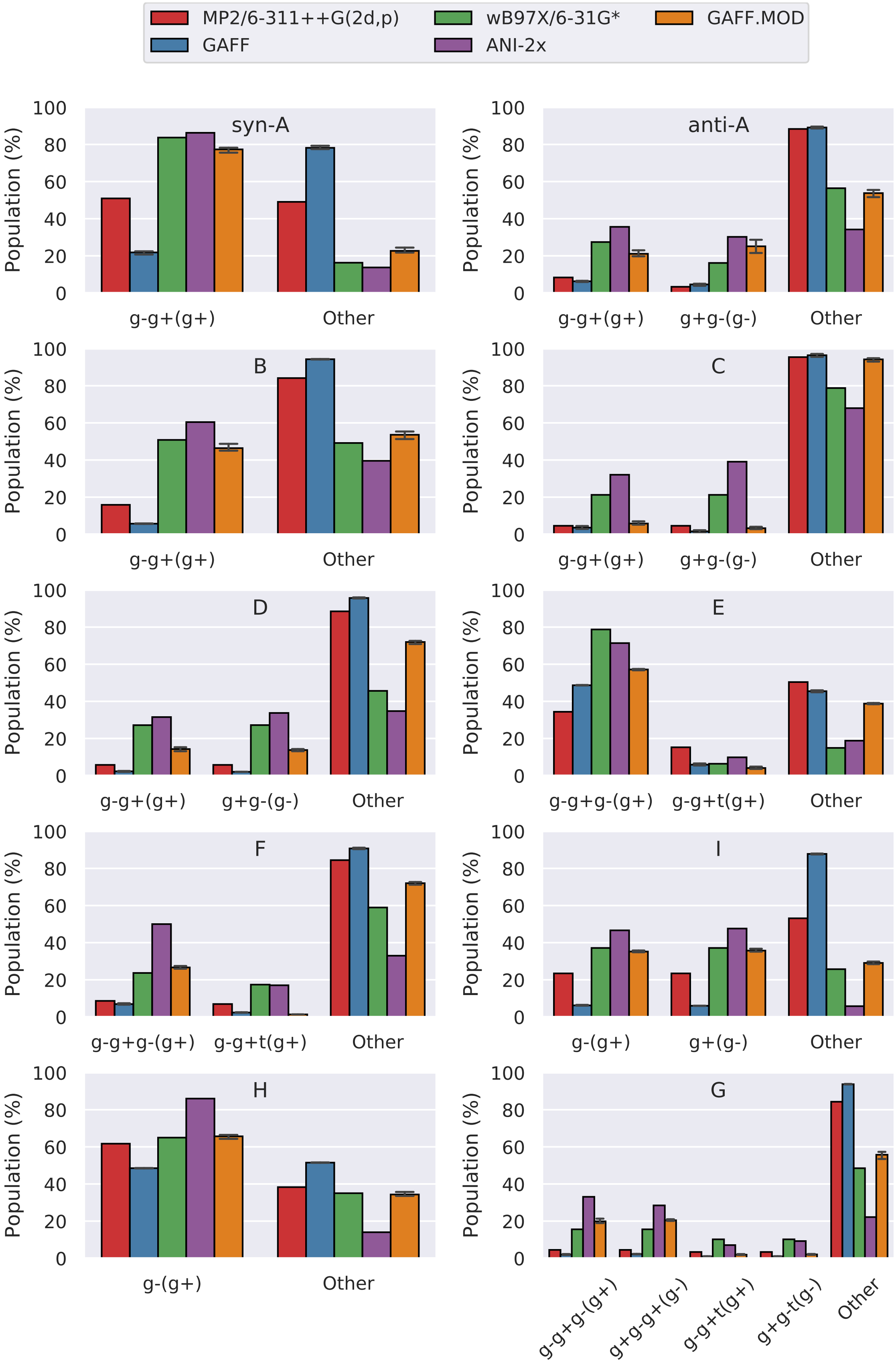

Figure S2: Populations in the gas phase of the conformers with IMHBs.

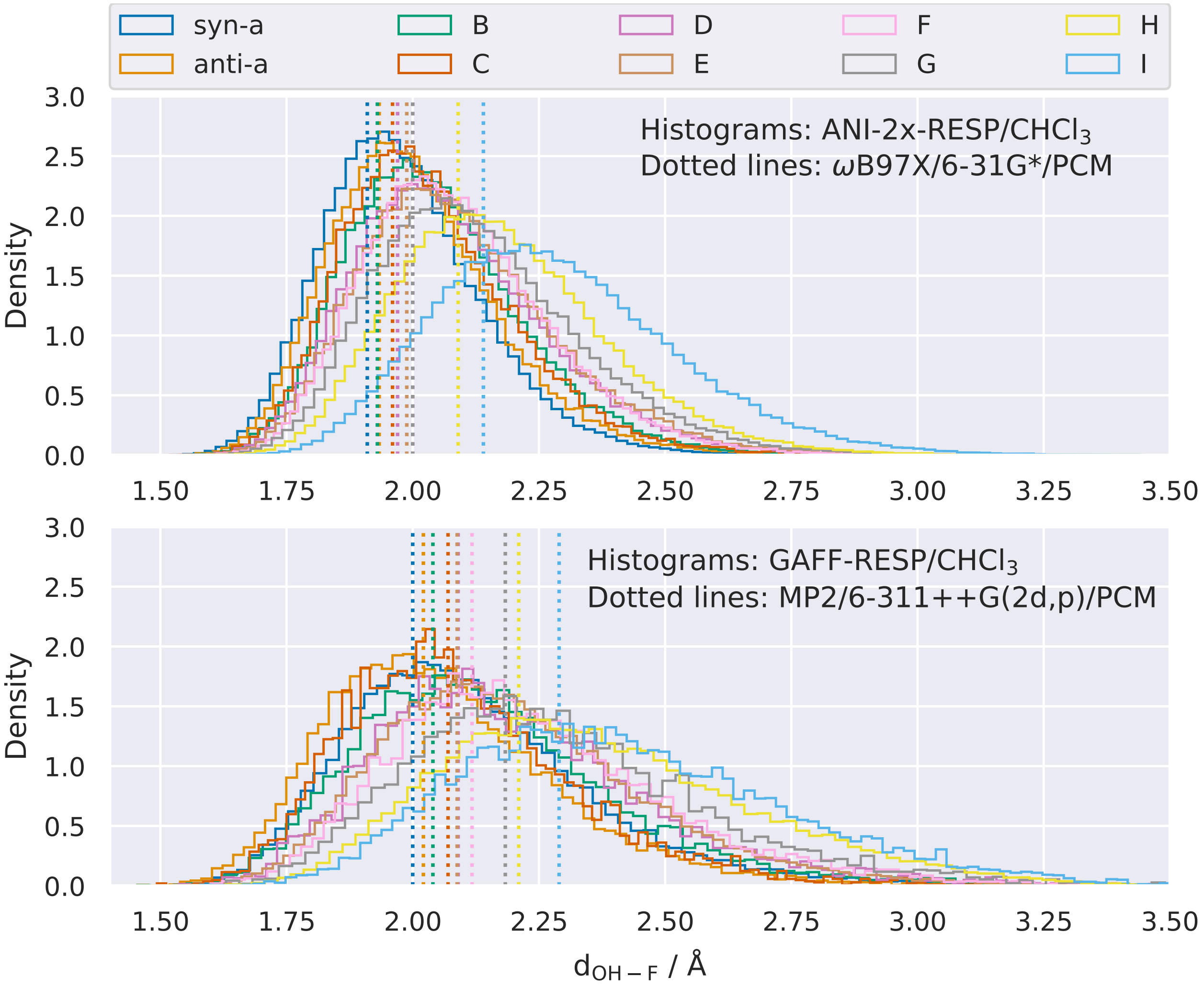

Figure S3: Top panel: Distributions of the hydrogen bond (HB) lengths as obtained from the ANI-2x-RESP/$CHCl_3$ MD simulations (solid lines), and HB lengths of the geometries optimized at ωB97X/6-31G*/PCM (dashed lines). Bottom panel: Distributions of the hydrogen bond (HB) lengths as obtained from the GAFF-RESP/$CHCl_3$ MD simulations (solid lines), and HB lengths of the geometries optimized at MP2/6-311++G(2d,p)/PCM (dashed lines). Only conformers with IMHBs are represented.

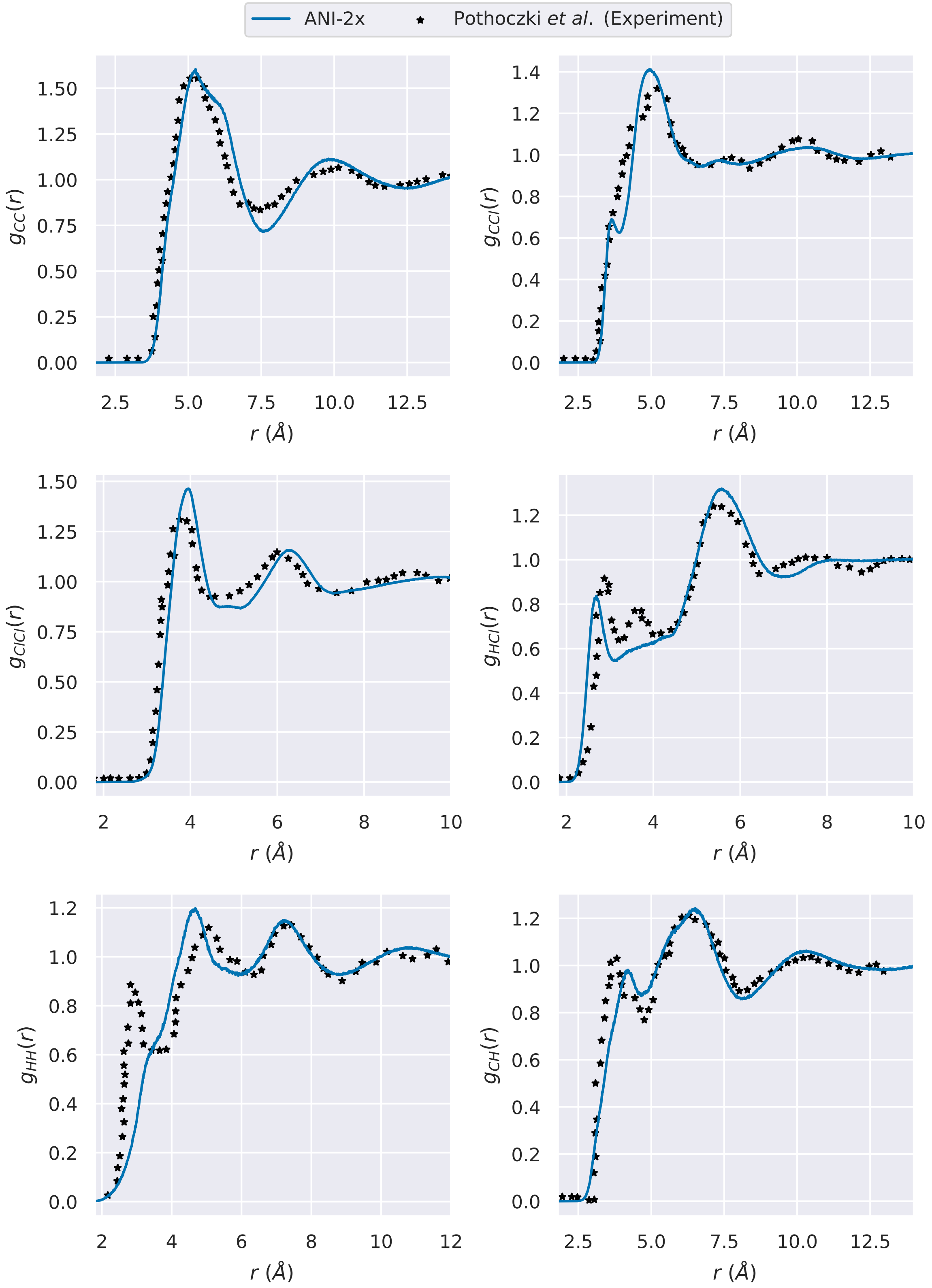

Figure S4: Experimental and ANI-2x radial distribution functions (RDFs) of bulk chloroform. The experimental data is reproduced from Refs. 1 and 2.

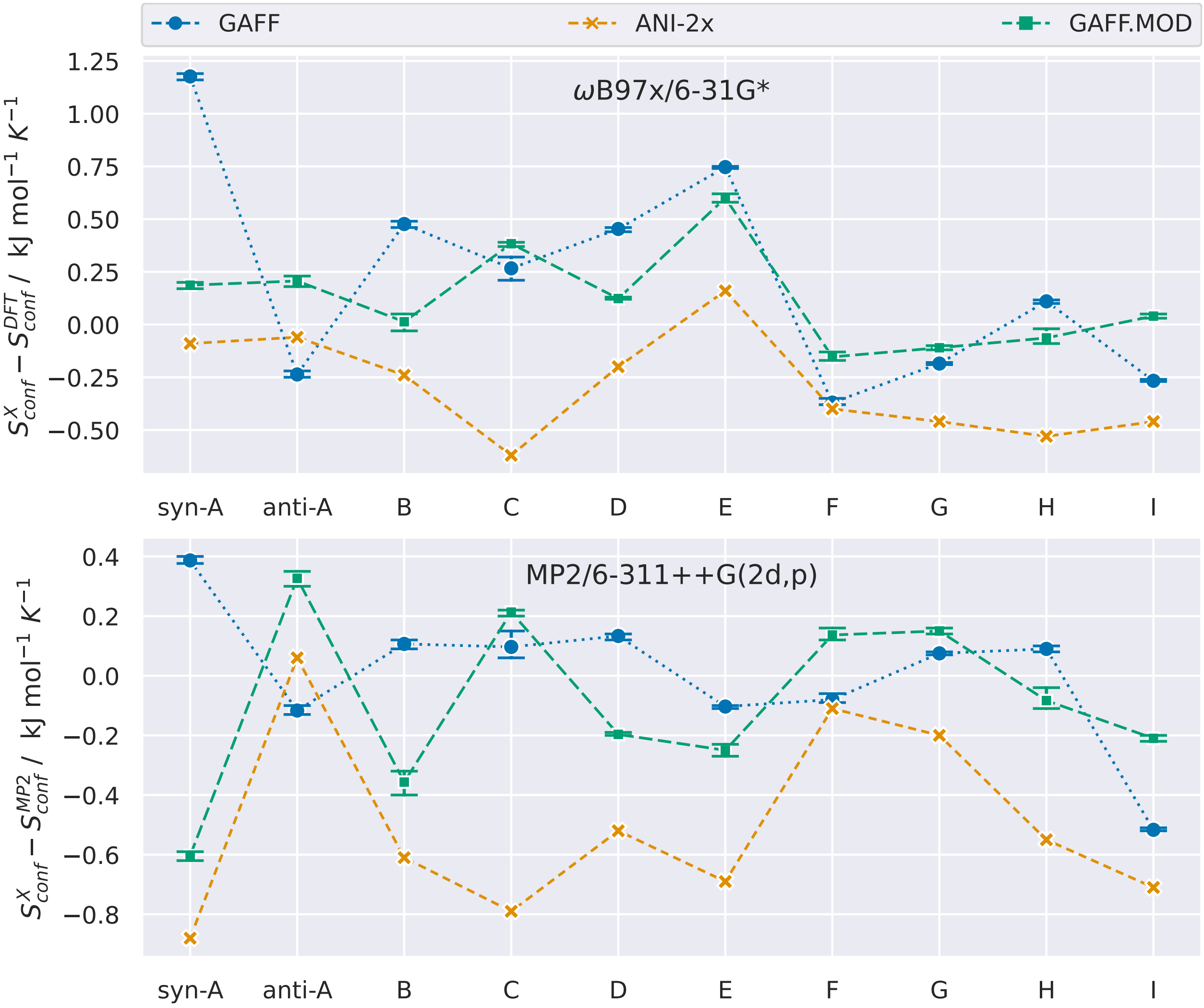

Figure S5: Gas-phase conformational entropy differences ($S_{conf} = -R\sum_i^{conf} p_i \ln[p_i]$) between the tested models and the reference QM levels. The QM references are ωb97X/6-31G* (top plot) and MP2/6-311++G(2d,p) (bottom plot).

References

(1) Pothoczki, S.; Temleitner, L.; Kohara, S.; Jóvári, P.; Pusztai, L. The Liquid Structure of Haloforms CHCl3 and CHBr3. *J. Phys.: Condens. Matter* **2010**, *22*, 404211.

(2) Yin, C.-C.; Li, A. H.-T.; Chao, S. D. Liquid Chloroform Structure From Computer Simulation With a Full Ab Initio Intermolecular Interaction Potential. *J. Chem. Phys.* **2013**, *139*, 194501.
